# Role of feeding specialization in taste receptor loss: insights from sweet and umami receptor evolution in Carnivora

**DOI:** 10.1093/chemse/bjac033

**Published:** 2022-11-22

**Authors:** Mieczyslaw Wolsan, Jun J Sato

**Affiliations:** Museum and Institute of Zoology, Polish Academy of Sciences, Wilcza 64, 00-679 Warszawa, Poland; Department of Biotechnology, Fukuyama University, Higashimura-cho, Aza, Sanzo, 985-1, Fukuyama 729-0292, Japan

**Keywords:** extragustatory function, feeding behavior, feeding ecology, gustation, loss of function, nonadaptive convergence

## Abstract

Controversy and misunderstanding surround the role of feeding specialization in taste receptor loss in vertebrates. We refined and tested the hypothesis that this loss is caused by feeding specializations. Specifically, feeding specializations were proposed to trigger time-dependent process of taste receptor loss through deprivation of benefit of using the receptor’s gustatory function. We propose that this process may be accelerated by abiotic environmental conditions or decelerated/stopped because of extragustatory functions of the receptor’s protein(s). As test case we used evolution of the sweet (TAS1R2+TAS1R3) and umami (TAS1R1+TAS1R3) receptors in Carnivora (dogs, cats, and kin). We predicted these receptors’ absence/presence using data on presence/absence of inactivating mutations in these receptors’ genes and data from behavioral sweet/umami preference tests. We identified 20 evolutionary events of sweet (11) or umami (9) receptor loss. These events affected species with feeding specializations predicted to favor sweet/umami receptor loss (27 and 22 species, respectively). All species with feeding habits predicted to favor sweet/umami receptor retention (11 and 24, respectively) were found to retain that receptor. Six species retained the sweet (5) or umami (1) receptor despite feeding specialization predicted to favor loss of that receptor, which can be explained by the time dependence of sweet/umami receptor loss process and the possible decelerating effect of TAS1R extragustatory functions so that the sweet/umami receptor process is ongoing in these species. Our findings support the idea that feeding specialization leads to taste receptor loss and is the main if not only triggering factor for evolutionary loss of taste receptors.

## Introduction

The vertebrate oral cavity is equipped with epithelial specializations containing taste-signaling molecules that interact with sweet-, umami-, bitter-, salty-, and sour-tasting compounds (Roura and Foster 2018; Töle et al. 2019). This chemosensory system of gustation serves evaluation of food quality, enabling nutritionally meaningful decisions. Although efficient gain of nutritious compounds and avoidance of harmful ones are essential for survival and reproduction, loss of taste receptors is widespread and has been reported from fishes (e.g., Liu et al. 2017), amphibians (e.g., Zhong et al. 2021), reptiles (e.g., Feng and Liang 2018), birds (e.g., Zhao et al. 2015), and mammals including humans (e.g., Go et al. 2005; Feng et al. 2014; Wolsan and Sato 2020).

While convergent trait changes are often adaptive, loss of taste receptors across vertebrates is nonadaptive and reflects release from functional constraint (Go et al. 2005; Jiang et al. 2012a; Hu et al. 2017; Tarusawa and Matsumura 2020; Wolsan and Sato 2020). A straightforward candidate cause of this release is evolutionary change leading to the restriction in diet contents that deprives of the benefit of using the receptor’s gustatory function. This potential cause was expressly addressed by Jiang et al. (2012a), who proposed that “loss of taste receptor function in mammals is […] directly related to feeding specializations.” Strictly, loss of a receptor’s function cannot be related “directly” to feeding specialization because intervening events such as relaxation of purifying selection on a gene expressed in that receptor, occurrence of a loss-of-function mutation in that gene, and fixation of that mutation need to take place to eventually lead to loss of that receptor’s function in a lineage. However, causal relation between feeding specializations and loss of taste receptors in mammals and other vertebrates seems likely because feeding specialization restricts diet composition. Nevertheless, Zhao and Zhang (2012) pointed to mismatches between feeding specializations and taste receptor presence/absence in mammals and birds and concluded that Jiang et al.’s (2012a) hypothesis is unwarranted. The role of feeding specialization in taste receptor loss has been debated since then and is currently subject of controversy and misunderstanding (Jiang et al. 2012b; Feng and Zhao 2013; Liu et al. 2016; Feng and Liang 2018; Jiao et al. 2021; Zhong et al. 2021).

Here, we refined and tested Jiang et al.’s (2012a) hypothesis using as test case the evolution of the sweet and umami taste receptors in the mammalian order Carnivora (dogs, cats, and relatives). Specifically, we tested whether loss of these receptors in carnivorans could have been driven by feeding specialization. We chose this test case because (i) the sweet and umami receptors share a common subunit, their genetic basis is well defined, and their gustatory function is relatively well understood (Roura and Foster 2018; Töle et al. 2019); (ii) loss of either or both receptors has been reported from multiple carnivoran species (Li et al. 2005, 2009, 2010; Zhao et al. 2010; Jiang et al. 2012a; Sato and Wolsan 2012; Hu et al. 2017; Tarusawa and Matsumura 2020; Wolsan and Sato 2020); (iii) carnivorans exhibit broad range of feeding specializations (Wilson and Mittermeier 2009, 2014); and (iv) carnivoran evolution, feeding ecology, and feeding behavior are relatively well known (Goswami 2010). We performed the test by confronting the inferred absence/presence of these receptors with various kinds of feeding habits and modes.

The sweet and umami taste receptors are TAS1R heterodimers, TAS1R2+TAS1R3 (Nelson et al. 2001) and TAS1R1+TAS1R3 (Li et al. 2002; Nelson et al. 2002), respectively. Loss of either TAS1R protein in either receptor causes loss of that receptor (Nelson et al. 2001, 2002; Li et al. 2002; Zhao et al. 2003). Because the TAS1R1, TAS1R2, and TAS1R3 proteins are encoded, respectively, by the *TAS1R1*, *TAS1R2*, and *TAS1R3* genes, loss of the *TAS1R1* gene causes loss of the umami receptor, loss of the *TAS1R2* gene causes loss of the sweet receptor, and loss of the *TAS1R3* gene causes loss of both receptors. This enables prediction of absence or presence of these receptors from absence or presence of *TAS1R* genes, which in turn can be predicted from presence or absence, respectively, of inactivating mutations that convert functional genes into nonfunctional pseudogenes. These mutations include easily recognizable start codon and nonsense substitutions and frameshift indels. While start codon mutations prevent gene translation, nonsense and frameshift mutations introduce stop codons that disrupt the open reading frame, causing premature termination of translation of nucleic acids into proteins. Some nonframeshift indels may also cause pseudogenization (e.g., an insertion that contains a stop codon or a deletion that removes a pivotal part of the gene).

While taste receptor pseudogenes are good indicators of the receptor’s inactivation, apparently intact taste receptor genes may not necessarily reflect the receptor’s full function. This is because protein dysfunction may also result from a regulatory mutation that abolishes gene expression or an unapparent coding-region missense mutation that causes crucial change in protein structure (Albalat and Cañestro 2016). It is of note that Jiao et al. (2021) postulated loss of the sweet taste receptor despite apparently intact *TAS1R2* and *TAS1R3* in insectivorous bats based on their indifference toward sucrose, fructose, and glucose. However, the TAS1R2+TAS1R3 heterodimer of these bats responded to an artificial sweetener, neohesperidin dihydrochalcone, and possibly may respond to carbohydrates contained in insects that were not tested by Jiao et al. (2021), e.g., trehalose, the principal sugar circulating in insect hemolymph (Gillott 2005). Moreover, given the time that has elapsed since the purported convergent loss of the sweet receptor from insectivorous bats (~20 to ~50 million years depending on the clade—Jiao et al. 2021), lack of any recognizable inactivating mutations in the insectivorous bat *TAS1R2* is unexpected. For comparison, during ~18 and ~20 million years following convergent loss of the sweet receptor from otarioids and phocids respectively, the pinniped *TAS1R2* has accumulated 2–7 recognizable inactivating mutations in each extant species separately (Wolsan and Sato 2020). While clarification of the sweet receptor condition in insectivorous bats needs further research, inferring the presence of taste receptors from apparently intact taste receptor genes requires caution and should ideally be confirmed by behavioral experiments.

## Methods

### Collection of DNA and behavioral data

We checked carnivoran *TAS1R*s for harboring potential inactivating mutations using multiple DNA sequence alignments generated with muscle in mega 6.0 (Tamura et al. 2013) and, where needed, manually adjusted according to similarity and parsimony criteria. These sequences were generated (Supplementary Table S1) or retrieved from DDBJ/ENA/GenBank (Supplementary Table S2). In addition, we used published data on presence/absence of *TAS1R* inactivating mutations in Carnivora (Li et al. 2005, 2009, 2010; Shi and Zhang 2006; Zhao et al. 2010; Jiang et al. 2012a; Sato and Wolsan 2012; Hu et al. 2017; Tarusawa and Matsumura 2020; Wolsan and Sato 2020) and published data from behavioral sweet/umami preference tests (Beauchamp et al. 1977; Ferrell 1984; Li et al. 2009; Jiang et al. 2012a, 2014).

The generated DNA sequences were deposited in DDBJ/ENA/GenBank with accession numbers LC654483 to LC654503 and LC727905 to LC727975. These sequences were obtained as follows. First, genomic DNA was isolated from samples listed in Supplementary Table S1 using a phenol–chloroform method or the DNeasy Blood and Tissue Kit (Qiagen). Next, polymerase chain reaction (PCR) amplification of a targeted gene region was carried out using the KOD FX Neo polymerase (Toyobo) and a combination of primers (Supplementary Tables S3–S20). The PCR reaction mixture contained 2× PCR buffer for KOD FX Neo, 0.4 mM dNTP mix, 1.0 U of KOD FX Neo, 0.3 μM of each primer, and 0.1–0.2 μg of genomic DNA in a total volume of 50 μl. PCR reactions were conducted on an automated thermal cycler (model PC 808, Astec) under the following conditions: a 1-min denaturation period at 94°C followed by 35 cycles of denaturation at 98°C for 10 s, annealing at 50°C for 30 s, and extension at 68°C for a time duration according to the anticipated length of the amplicon (basically 90 s); this was followed by an extension period at 68°C for 10 min. Then, each target PCR product with an anticipated length was excised from the postelectrophoresis low-melting-point agarose gel, purified by a phenol–chloroform method, and precipitated with ethanol. Finally, sequencing was performed by the Sanger method using the BigDye Terminator v3.1 Cycle Sequencing Kit (Applied Biosystems). Sequence data were acquired on an ABI3130 automated sequencer (Applied Biosystems).

### Categorization of feeding habits and modes

Species were classified into 9 feeding habit and 2 feeding mode categories. The feeding habit categories comprised cancri–piscivores (annual diets of >75% crustaceans and fish, with >50% crustaceans); carni–piscivores (annual diets of >80% tetrapods and fish, with 55–70% tetrapods and 25–35% fish); carnivores (annual diets of >80% tetrapods); herbivores (annual diets of >80% bamboo); insecti–carnivores (annual diets of >85% insects and tetrapods, with 50–65% insects and 30–45% tetrapods); insecti–frugivores (annual diets of >90% insects and fruits, with <60% insects and <60% fruits); insectivores (annual diets of >80% insects); molluscivores (annual diets of >75% molluscs); omnivores (annual diets of tetrapods, other animals, and plants, with <80% tetrapods, <60% other animals, and <70% plants); pisci–molluscivores (annual diets of >75% fish and molluscs, with 35–55% fish and 35–55% molluscs), and piscivores (annual diets of ≥60% fish). The feeding mode categories comprised species that process food orally or those that swallow food whole. Data on the feeding habits and modes derived from Wilson and Mittermeier (2009, 2014) and Pauly et al. (1998).

### Predictions for hypothesis testing

Sweet taste is elicited by sugars and other compounds associated with carbohydrate sources (Roura and Foster 2018; Töle et al. 2019). Carbohydrates are generally abundant in plants but relatively scarce in animals (Alén 2018) with notable exception of insects, particularly phytophagous ones, which contain and may secrete sugars, often at high concentration (Gillott 2005). Therefore in line with the tested hypothesis, we predict that the sweet taste receptor will be present in herbivores, insecti–frugivores, insectivores, insecti–carnivores, and omnivores but absent from carnivores, piscivores, carni–piscivores, molluscivores, pisci–molluscivores, and cancri–piscivores.

Umami taste is evoked by l-amino acids (Ikeda 1909; Nelson et al. 2002) and considerably enhanced by purine 5ʹ-nucleotides (Kuninaka 1960; Yoshii et al. 1986). These enhancers are abundant in muscles and other tissues of tetrapods and insects but relatively scarce in living or fresh fish, molluscs, crustaceans, and bamboo (Arai and Saito 1961; Arai 1966; van Waarde 1988; Yamaguchi and Ninomiya 2000; Kurihara 2009; Kaneko et al. 2014). Moreover, insect hemolymph has very high amino acid content (Klowden 2013). Therefore in accordance with the tested hypothesis, we predict that the umami taste receptor will be present in carnivores, carni–piscivores, insecti–carnivores, insectivores, insecti–frugivores, and omnivores but absent from herbivores, piscivores, molluscivores, pisci–molluscivores, and cancri–piscivores.

Food swallowed whole without chewing may be untasted. Therefore, we predict that the sweet and umami taste receptors will be absent from species with this mode of feeding, regardless of the feeding habit.

## Results

**Fig. 1.**
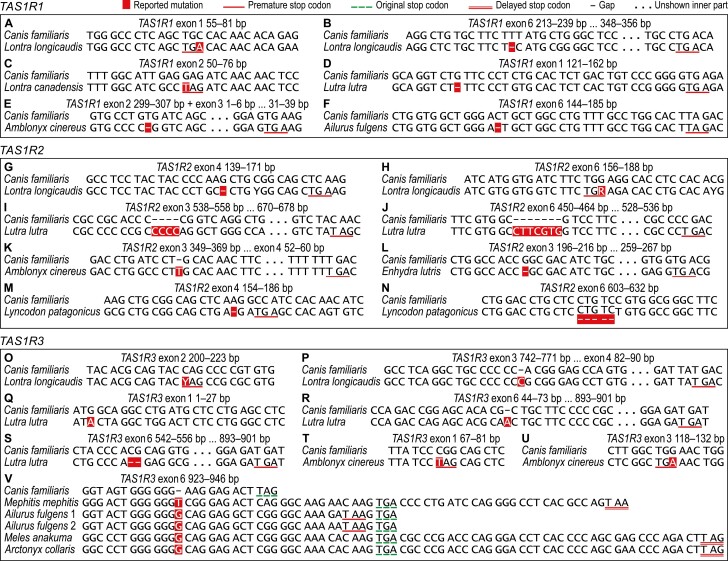
DNA sequence alignments showing the start codon, nonsense, and frameshift mutations found in this study. (A) Homozygous substitution of C to A at 69 bp of *TAS1R1* exon 1 in *Lontra longicaudis* resulting in a premature homozygous stop codon (67–69 bp). (B) 1-bp deletion at 226 bp of *TAS1R1* exon 6 in *Lontra longicaudis* resulting in multiple premature homozygous stop codons with the first one at 352–354 bp. (C) Homozygous substitution of G to T at 62 bp of *TAS1R1* exon 2 in *Lontra canadensis* resulting in a premature homozygous stop codon (62–64 bp). (D) 1-bp deletion at 129 bp of *TAS1R1* exon 1 in *Lutra lutra* resulting in multiple premature homozygous stop codons with the first one at 158–160 bp of exon 1. (E) 1-bp deletion at 306 bp of *TAS1R1* exon 2 in *Amblonyx cinereus* resulting in multiple premature homozygous stop codons with the first one at 35–37 bp of exon 3. (F) 1-bp deletion at 157 bp of *TAS1R1* exon 6 in *Ailurus fulgens* resulting in multiple premature homozygous stop codons with the first one at 181–183 bp. (G) 1-bp deletion at 156 bp of *TAS1R2* exon 4 in *Lontra longicaudis* resulting in multiple premature homozygous stop codons with the first one at 167–169 bp of exon 4. (H) Heterozygous substitution of G to A at 173 bp of *TAS1R2* exon 6 in *Lontra longicaudis* resulting in a premature heterozygous stop codon (TGA at 171–173 bp). (I) 4-bp insertion between 547 and 548 bp of *TAS1R2* exon 3 in *Lutra lutra* resulting in multiple premature homozygous stop codons with the first one at 675–677 bp of exon 3. (J) 7-bp insertion between 457 and 458 bp of *TAS1R2* exon 6 in *Lutra lutra* resulting in multiple premature homozygous stop codons with the first one at 533–535 bp. (K) 1-bp insertion between 359 and 360 bp of *TAS1R2* exon 3 in *Amblonyx cinereus* (LC654484 and JN130352) resulting in multiple premature homozygous stop codons with the first one at 57–59 bp of exon 4. (L) 1-bp deletion at 205 bp of *TAS1R2* exon 3 in *Enhydra lutris* resulting in multiple premature homozygous stop codons with the first one at 263–265 bp of exon 3. (M) 1-bp deletion at 170 bp of *TAS1R2* exon 4 in *Lyncodon patagonicus* resulting in multiple premature homozygous stop codons with the first one at 173–175 bp of exon 4. (N) 5-base deletion at 615–619 bp of *TAS1R2* exon 6 in *Lyncodon patagonicus* not resulting in a premature stop codon. (O) Heterozygous substitution of C to T at 212 bp of *TAS1R3* exon 2 in *Lontra longicaudis* resulting in a premature heterozygous stop codon (TAG at 212–214 bp). (P) 1-bp insertion between 758 and 759 bp of *TAS1R3* exon 3 in *Lontra longicaudis* resulting in multiple premature homozygous stop codons with the first one at 87–89 bp of exon 4. (Q) Homozygous substitution of G to A in the start codon of *TAS1R3* in *Lutra lutra* (nearest downstream in-frame ATG sequence is at 85–87 bp of exon 1). (R) 1-bp insertion between 60 and 61 bp of *TAS1R3* exon 6 in *Lutra lutra* resulting in a premature homozygous stop codon at 898–900 bp. (S) 2-bp deletion at 549 and 550 bp of *TAS1R3* exon 6 in *Lutra lutra* resulting in a premature homozygous stop codon at 898–900 bp. (T) Homozygous substitution of C to T at 73 bp of *TAS1R3* exon 1 in *Amblonyx cinereus* resulting in a premature homozygous stop codon (73–75 bp). (U) Homozygous substitution of G to A at 126 bp of *TAS1R3* exon 3 in *Amblonyx cinereus* resulting in a premature homozygous stop codon (124–126 bp). (V) 1-bp insertion between 934 and 935 bp of *TAS1R3* exon 6 in *Mephitis mephitis*, *Ailurus fulgens* (1, LC654495; 2, LNAC01000817), *Meles anakuma*, and *Arctonyx collaris* resulting in a premature (*Ailurus fulgens*) or delayed (remaining species) homozygous stop codon; note that the arctoid *TAS1R3* exon 6 is 5 codons longer than the canid one (this study; Wolsan and Sato 2020). Base-pair position numbering refers to the aligned *Canis familiaris* sequence, starts from the 5ʹ end of each exon separately, and is from left to right. Codons in the correct open reading frame are separated by spaces.

**Fig. 2.**
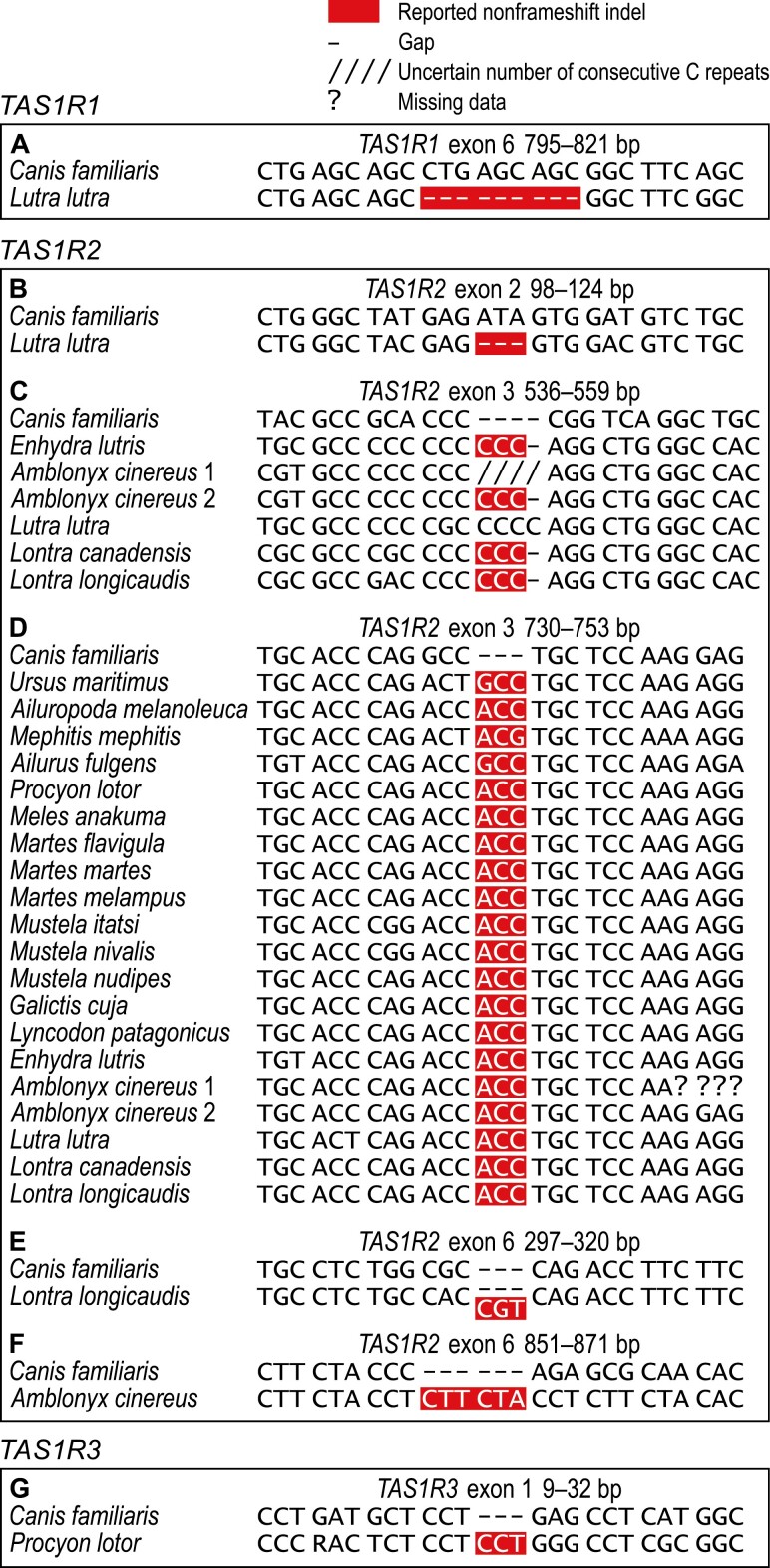
DNA sequence alignments showing the nonframeshift indels found in this study. (A) 9-bp deletion at 804–812 bp of *TAS1R1* exon 6 in *Lutra lutra*. (B) 3-bp deletion at 110–112 bp of *TAS1R2* exon 2 in *Lutra lutra*. (C) 3-bp insertion between 547 and 548 bp of *TAS1R2* exon 3 in *Enhydra lutris*, *Amblonyx cinereus* (1, LC654484; 2, JN130352), *Lontra canadensis*, and *Lontra longicaudis*; note that *Lutra lutra* has instead a 4-bp frameshift insertion, which is reported in Fig. 1I. (D) 3-bp insertion between 741 and 742 bp of *TAS1R2* exon 3 in all arctoids for which this part was sequenced (for *Amblonyx cinereus*: 1, LC654484; 2, JN130352); note that the ACC sequence prevails and is also consistently present in pinnipeds (Wolsan and Sato 2020: Fig. S2), which suggests that the GCC sequence in *Ailurus fulgens* and *Ursus maritimus* has evolved by a secondary substitution of A to G and the ACG sequence in *Mephitis mephitis* by secondary substitution of C to G. (E) 3-base insertion between 308 and 309 bp of *TAS1R2* exon 6 in *Lontra longicaudis*. (F) 6-bp insertion between 859 and 860 bp of *TAS1R2* exon 6 in *Amblonyx cinereus*. (G) 3-bp insertion between 20 and 21 bp of *TAS1R3* exon 1 in *Procyon lotor*. Base-pair position numbering refers to the aligned *Canis familiaris* sequence, starts from the 5ʹ end of each exon separately, and is from left to right. Codons in the correct open reading frame are separated by spaces.

Examination of the DNA sequences determined here and those obtained from DDBJ/ENA/GenBank revealed 30 species-specific or shared potential inactivating mutations. We first report a start codon substitution in *TAS1R3* of *Lutra lutra* (Fig. 1Q); nonsense substitutions in all *TAS1R*s of *Lontra longicaudis* (Fig. 1A,H,O), *TAS1R1* of *Lontra canadensis* (Fig. 1C), and *TAS1R3* of *Amblonyx cinereus* (Fig. 1T,U); frameshift indels in all *TAS1R*s of *Lontra longicaudis* (Fig. 1B,G,P), *TAS1R2* and *TAS1R3* of *Lutra lutra* (Fig. 1I,J,R,S), *TAS1R1* of *Amblonyx cinereus* (Fig. 1E), *TAS1R2* of *Enhydra lutris* (Fig. 1L) and *Lyncodon patagonicus* (Fig. 1M,N), and *TAS1R3* of *Arctonyx collaris*, *Meles anakuma*, *Ailurus fulgens*, and *Mephitis mephitis* (Fig. 1V); and nonframeshift indels in *TAS1R1* and *TAS1R2* of *Lutra lutra* (Fig. 2A,B,D), *TAS1R3* of *Procyon lotor* (Fig. 2G), and *TAS1R2* of *Lontra longicaudis* (Fig. 2C–E), *Lontra canadensis* (Fig. 2C,D), *Amblonyx cinereus* (Fig. 2C,D,F), *Enhydra lutris* (Fig. 2C,D), and 12 other musteloids and 2 ursids (Fig. 2D). We also confirm a 1-bp insertion in *TAS1R2* of *Amblonyx cinereus* (Fig. 1K), which was first reported from JN130352 by Jiang et al. (2012a); and 1-bp deletions in *TAS1R1* of *Lutra lutra* (Fig. 1D) and *Ailurus fulgens* (Fig. 1F), which were first reported from other individuals of these species by Tarusawa and Matsumura (2020) and Hu et al. (2017), respectively.

## Discussion

### Assessing the effect of mutations found in this study

The start codon mutation in *Lutra lutra* (Fig. 1Q) is predicted to prevent *TAS1R3* from being translated. Defects in the coding sequence inflicted by the referred illustrated mutations predict that the following *TAS1R*s are pseudogenes: all *TAS1R*s of *Lontra longicaudis* (Fig. 1A,B,G,P), *Lutra lutra* (Fig. 1D,I,J,R,S), and *Amblonyx cinereus* (Fig. 1E,K,T,U); *TAS1R1* of *Lontra canadensis* (Fig. 1C) and *Ailurus fulgens* (Fig. 1F); and *TAS1R2* of *Enhydra lutris* (Fig. 1L) and *Lyncodon patagonicus* (Fig. 1M). The *TAS1R2* and *TAS1R3* nonsense substitutions in *Lontra longicaudis* (Fig. 1H,O) and the *TAS1R2* 5-bp deletion in *Lyncodon patagonicus* (Fig. 1N) are heterozygous and therefore only potentially inactivating, but these *TAS1R*s also harbor already mentioned homozygous frameshift mutations that predict them to be pseudogenes.

*Ailurus fulgens* senses sweet taste-eliciting compounds (Li et al. 2009) despite its *TAS1R3* G insertion (Fig. 1V), which indicates that this mutation is not inactivating. Therefore, we infer that the convergent mutation in *Arctonyx collaris* and *Meles anakuma* and the orthologous T insertion in *Mephitis mephitis* (Fig. 1V) are not inactivating either. Our conclusion that *TAS1R3* of *Ailurus fulgens* is a functional gene concurs with that of Hu et al. (2017), who reported this species’ *TAS1R3* as “intact” based on LNAC01000817, although they did not mention that the G insertion is present in this sequence (Fig. 1V: *Ailurus fulgens* 2).

Ability to sense sweet taste-eliciting compounds by *Ailurus fulgens* and *Ailuropoda melanoleuca* (Li et al. 2009; Jiang et al. 2014) proves that the 3-bp insertion in their *TAS1R2* (Fig. 2D) is not inactivating. Therefore, we conclude that the same or orthologous insertion in other arctoids (Fig. 2D) is not inactivating either. We also assume that the remaining nonframeshift indels found in this study (Fig. 2A–C,E–G) are not inactivating because they are similar to the arctoid insertion (only 1–3 codons long and not causing a shift in the open reading frame).

### Evolution of the sweet and umami taste receptors in Carnivora versus feeding habits and modes

**Fig. 3.**
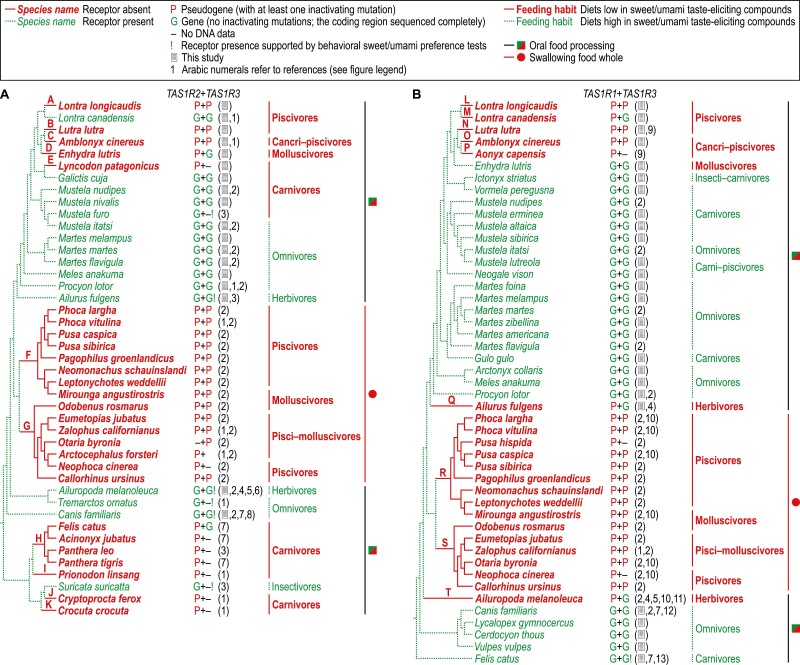
Evolution of the sweet (A) and umami (B) taste receptors in Carnivora in relation to feeding habits and modes. The events of loss of these receptors (A to T) are inferred from phylogenetic placement of *TAS1R* inactivating mutations (Tables 1 and 2). Not shown are species with no or incomplete DNA data that prevented conclusion about sweet/umami receptor absence/presence (for all species, see Supplementary Fig. S1). Phylogenies are compiled from Koepfli et al. (2008), Sato et al. (2012), Wolsan and Sato (2020), Pagès et al. (2008), Lindblad-Toh et al. (2005), Johnson et al. (2006), and Eizirik et al. (2010). References: 1, Jiang et al. (2012a); 2, Wolsan and Sato (2020); 3, Li et al. (2009); 4, Hu et al. (2017); 5, Li et al. (2010); 6, Jiang et al. (2014); 7, Li et al. (2005); 8, Ferrell (1984); 9, Tarusawa and Matsumura (2020); 10, Sato and Wolsan (2012); 11, Zhao et al. (2010); 12, Shi and Zhang (2006); 13, Beauchamp et al. (1977).

The *TAS1R* inactivating mutations discovered in Carnivora by this and previous studies predict absence of the sweet receptor from 27 evaluated species (Fig. 3A) and the umami receptor from 22 evaluated species (Fig. 3B). We mapped these mutations on carnivoran phylogeny and found evidence of 11 separate evolutionary events of loss of the sweet receptor (Table 1) and 9 separate evolutionary events of loss of the umami receptor (Table 2). No inactivating mutations in the completely sequenced coding regions of *TAS1R*s confirmed by this and previous studies and preference toward sweet/umami taste-eliciting compounds corroborated by behavioral investigations let to predict that 16 evaluated species have retained the sweet receptor (Fig. 3A) and 25 evaluated species have retained the umami receptor (Fig. 3B).

**Table 1.** Mutations hypothesized to cause evolutionary events of loss of the sweet taste receptor in Carnivora.

Event^a^	Taxon affected	Mutation’s genomic location		Mutation^b^	Reference
		Gene	Exon		
A	*Lontra longicaudis*	*TAS1R2*	Exon 4	1-bp deletion	Fig. 1G
		*TAS1R3*	Exon 3	1-bp insertion	Fig. 1P
B	*Lutra lutra*	*TAS1R2*	Exon 3	4-bp insertion^c^	Fig. 1I
			Exon 6	7-bp insertion	Fig. 1J
		*TAS1R3*	Exon 1	Start codon homozygous substitution	Fig. 1Q
			Exon 6	1-bp insertion	Fig. 1R
				2-bp deletion	Fig. 1S
C	*Amblonyx cinereus*	*TAS1R2*	Exon 3	1-bp insertion	Fig. 1K; Jiang et al. 2012a: Fig. 1B
		*TAS1R3*	Exon 1	Homozygous nonsense substitution	Fig. 1T
			Exon 3	Homozygous nonsense substitution	Fig. 1U
D	*Enhydra lutris*	*TAS1R2*	Exon 3	1-bp deletion	Fig. 1L
E	*Lyncodon patagonicus*	*TAS1R2*	Exon 4	1-bp deletion	Fig. 1M
F	Phocidae	*TAS1R2*	Exon 6	Homozygous nonsense substitution	Wolsan and Sato 2020: mutation S15
		*TAS1R3*	Exon 3	1-bp deletion	Wolsan and Sato 2020: mutation US11
			Exon 6	1-bp deletion	Wolsan and Sato 2020: mutation US35
				1-bp deletion	Wolsan and Sato 2020: mutation US36
G	Otarioidea	*TAS1R2*	Exon 1	Start codon homozygous substitution	Wolsan and Sato 2020: mutation S1
			Exon 3	2-bp deletion	Wolsan and Sato 2020: mutation S12
			Exon 5	Homozygous nonsense substitution	Wolsan and Sato 2020: mutation S13
			Exon 6	1-bp deletion	Wolsan and Sato 2020: mutation S21
		*TAS1R3*	Exon 6	14-bp deletion	Wolsan and Sato 2020: mutation US30
H	Felidae	*TAS1R2*	Exon 3	247-bp deletion	Li et al. 2005: Fig. 1
I	*Prionodon linsang*	*TAS1R2*	Exon 2	1-bp insertion	Jiang et al. 2012a: Fig. 1B
				10-bp deletion	Jiang et al. 2012a: Fig. S6B
			Exon 4	14-bp insertion	Jiang et al. 2012a: Fig. S6C
			Exon 5	20-bp deletion	Jiang et al. 2012a: Fig. S6D
				2-bp deletion	Jiang et al. 2012a: Fig. S6E
			Exon 6	1-bp deletion	Jiang et al. 2012a: Fig. S6F
				28-bp insertion	Jiang et al. 2012a: Fig. S6G
				1-bp deletion	Jiang et al. 2012a: Fig. S6H
J	*Cryptoprocta ferox*	*TAS1R2*	Exon 3	Homozygous nonsense substitution	Jiang et al. 2012a: Fig. 1B
			Exon 4	1-bp insertion	Jiang et al. 2012a: Fig. S5B
K	*Crocuta crocuta*	*TAS1R2*	Exon 2	1-bp deletion	Jiang et al. 2012a: Fig. 1B

^a^All events are illustrated in Fig. 3A.

^b^Mutations within the same exon are listed in order from the 5ʹ to 3ʹ end.

^c^Other otters (*Lontra longicaudis*, *Lontra canadensis*, *Amblonyx cinereus*, *Enhydra lutris*) have instead a 3-bp (CCC) insertion, which suggests that the frameshift in *Lutra lutra* has effectively been caused by a 1-bp insertion, an extra repeat of C, rather than the 4-bp (CCCC) insertion (Fig. 2C).

**Table 2.** Mutations hypothesized to cause evolutionary events of loss of the umami taste receptor in Carnivora.

Event^a^	Taxon affected	Mutation’s genomic location		Mutation^b^	Reference
		Gene	Exon		
L	*Lontra longicaudis*	*TAS1R1*	Exon 1	Homozygous nonsense substitution	Fig. 1A
			Exon 6	1-bp deletion	Fig. 1B
		*TAS1R3*	Exon 3	1-bp insertion	Fig. 1P
M	*Lontra canadensis*	*TAS1R1*	Exon 2	Homozygous nonsense substitution	Fig. 1C
N	*Lutra lutra*	*TAS1R1*	Exon 1	1-bp deletion	Fig. 1D; Tarusawa and Matsumura 2020: Fig. S1
		*TAS1R3*	Exon 1	Start codon homozygous substitution	Fig. 1Q
			Exon 6	1-bp insertion	Fig. 1R
				2-bp deletion	Fig. 1S
O	*Amblonyx cinereus*	*TAS1R1*	Exon 2	1-bp deletion	Fig. 1E
		*TAS1R3*	Exon 1	Homozygous nonsense substitution	Fig. 1T
			Exon 3	Homozygous nonsense substitution	Fig. 1U
P	*Aonyx capensis*	*TAS1R1*	Exon 6	4-bp deletion	Tarusawa and Matsumura 2020: Fig. S2
Q	*Ailurus fulgens*	*TAS1R1*	Exon 6	1-bp deletion	Fig. 1F; Hu et al. 2017: Fig. 3
R	Phocidae	*TAS1R1*	Exon 2	41-bp deletion	Wolsan and Sato 2020: mutation U4
			Exon 3	4-bp insertion	Sato and Wolsan 2012: Fig. 1; Wolsan and Sato 2020: mutation U9
		*TAS1R3*	Exon 3	1-bp deletion	Wolsan and Sato 2020: mutation US11
			Exon 6	1-bp deletion	Wolsan and Sato 2020: mutation US35
				1-bp deletion	Wolsan and Sato 2020: mutation US36
S	Otarioidea	*TAS1R1*	Exon 2	1-bp deletion	Wolsan and Sato 2020: mutation U2
		*TAS1R3*	Exon 6	14-bp deletion	Wolsan and Sato 2020: mutation US30
T	*Ailuropoda melanoleuca*	*TAS1R1*	Exon 3	2-bp insertion	Li et al. 2010; Zhao et al. 2010; Sato and Wolsan 2012: Fig. 1; Hu et al. 2017: Fig. 3; Wolsan and Sato 2020
			Exon 6	4-bp deletion	Li et al. 2010; Zhao et al. 2010; Hu et al. 2017: Fig. 3; Wolsan and Sato 2020

^a^All events are illustrated in Fig. 3B.

^b^Mutations within the same exon are listed in order from the 5ʹ to 3ʹ end.

Confronting the inferred evolution of both receptors with feeding habits and modes (Fig. 3) shows that all of the events of sweet/umami receptor loss exclusively affect species with feeding specializations predicted to favor loss of the respective receptor. Namely, the sweet receptor loss events affect piscivores (events A, B, F, and G), cancri–piscivores (event C), molluscivores (events D, F, and G), carnivores (events E and H–K), pisci–molluscivores (event G), and species that swallow food whole (events F and G); while the umami receptor loss events affect piscivores (events L–N, R, and S), cancri–piscivores (events O and P), herbivores (events Q and T), molluscivores (events R and S), pisci–molluscivores (event S), and species that swallow food whole (events R and S). Moreover, all species with feeding habits predicted to favor sweet/umami receptor retention are inferred to retain the respective receptor. Namely, both herbivores and the sole insectivore are inferred to retain the sweet receptor; all carnivores and carni–piscivores and the sole insecti–carnivore are inferred to retain the umami receptor; and all omnivores are inferred to retain both receptors. All this is clearly consistent with the hypothesis that loss of taste receptors is caused by feeding specializations. However, seemingly contrary to this hypothesis, 6 species are inferred to retain the sweet or umami receptor despite the feeding specialization predicted to favor loss of that receptor. Namely, 1 piscivore (*Lontra canadensis*) and 4 carnivores (*Galictis cuja*, *Mustela nudipes*, *Mustela nivalis*, *Mustela furo*) are inferred to retain the sweet receptor (Fig. 3A), and 1 molluscivore (*Enhydra lutris*) is inferred to retain the umami receptor (Fig. 3B). We will further argue that these 6 exceptions are expected and do not contradict the hypothesized causal relationship between feeding specialization and taste receptor loss.

### Explanation for taste receptor retention despite a feeding specialization that favors the receptor’s loss

The hypothesis tested here assumes that feeding specialization deprives of the advantage of sensing a taste and therefore releases from selective pressure to maintain the receptor of that taste. This release may eventually result in fixation of a random inactivating mutation in the receptor’s gene and the resulting loss of integrity and consequently function of both the gene and the receptor. Therefore, a stochastic process is involved that continues over evolutionary time (Jiang et al. 2012b).

Although this hypothesis proposes feeding specialization to be the factor that triggers this process, the process itself may be accelerated, decelerated, or even stopped by other factors. The acceleration of taste receptor loss process can be caused by abiotic environmental conditions. For example, reduction of transduction of taste signal in cold water because of temperature sensitivity of TRPM5 (Talavera et al. 2005) and taste masking by high concentration of sodium in seawater (Ikeda 1909; Komata 1990) likely accelerated loss of taste receptors in pinnipeds (Sato and Wolsan 2012; Wolsan and Sato 2020) and penguins (Zhao et al. 2015) if the corresponding molecules had not been adjusted to the cold and marine environments fast enough.

In turn, the deceleration or stop of taste receptor loss process can be caused by selective pressure resulting from extragustatory functions of the taste receptor’ protein(s) because taste receptor proteins have multiple nongustatory functions (Roura and Foster 2018; Töle et al. 2019). For example, TAS1Rs have been reported to have, in addition to their gustatory function in the sweet and umami receptors, nongustatory functions in the brain (Ren et al. 2009), airways (Lee et al. 2014), gastrointestinal tract (Dyer et al. 2005; Bezençon et al. 2007; Jang et al. 2007; Margolskee et al. 2007; Steinert et al. 2011), pancreas (Nakagawa et al. 2009), testes and spermatozoa (Meyer et al. 2012; Mosinger et al. 2013), and other extraoral tissues (see, e.g., a review by Behrens et al. 2014) of mice, rats, and/or humans. That extragustatory functions of taste receptor proteins are capable to exert selective pressure is for example demonstrated by the maintaining of purifying selection on TAS1R1 despite loss of the umami receptor (by inactivation of TAS1R3) for ~4 million years in phocids and ~6 million years in otarioids, which we reported previously (Wolsan and Sato 2020). In turn, Feng et al. (2014) and Zhu et al. (2014) reported a plausible example of blockage of taste receptor loss process by extragustatory functions of taste receptor proteins. These authors discovered that cetaceans have lost all taste receptors except the salty one (potentially the epithelial sodium channel, ENaC) and pointed to extragustatory functions of ENaC proteins in sodium reabsorption across epithelia (Canessa et al. 1994) as the cause of this receptor’s retention. Note that loss of ENaC was demonstrated to be lethal to mice (Hummler and Beermann 2000).

The sweet/umami taste receptor evolution inferred here for clades containing species that have retained the respective receptor despite a feeding specialization that favors loss of that receptor well reflects the stochastic and time-dependent nature of taste receptor loss process with the possible decelerating effect of selective pressure arising from extragustatory functions of taste receptor proteins. Figure 3 and Tables 1 and 2 show that loss of the sweet and umami receptors in the crown clade of otters (Lutrinae) has been achieved by convergent evolution with multiple separate loss events caused by different inactivating mutations. Specifically, both receptors have convergently been lost in the lineages of *Lontra longicaudis* (events A and L), *Lutra lutra* (events B and N), and *Amblonyx cinereus* (events C and O); the sweet receptor has also been convergently lost in the lineage of *Enhydra lutris* (event D) but is retained in the lineage of *Lontra canadensis*; and the umami receptor has also been convergently lost in the lineages of *Lontra canadensis* (event M) and *Aonyx capensis* (event P) but is retained in the lineage of *Enhydra lutris*. Judging from molecular dating estimates for lutrine divergences (Koepfli et al. 2008; Sato et al. 2009, 2012), event D is younger than ~5 million years; events B, C, and N are younger than ~3.5 million years; and events A, L, M, O, and P are younger than ~2.5 million years. The relative recency of these events concurs with small number of inactivating mutations (1–3) that have accumulated in each lutrine *TAS1R* pseudogene separately (Tables 1 and 2). Molecular dating has estimated that Lutrinae arose ~8–9 million years ago in the late Miocene (Yonezawa et al. 2007; Koepfli et al. 2008; Sato et al. 2009, 2012; Eizirik et al. 2010). The late Miocene fossil record of otters and otter-like mustelids, which exhibit adaptations indicative of feeding on fish, molluscs, or crustaceans (Morales and Pickford 2005; Pickford 2007; Haile-Selassie 2008; Peigné et al. 2008; Villier et al. 2011; Tseng et al. 2017; Wang et al. 2018), suggests that the switch to diets low in sweet and umami taste-eliciting compounds occurred in otters no later than at the origin of Lutrinae. This fossil-based conclusion concurs with the fact that all extant otters are piscivores, cancri–piscivores, or molluscivores (Wilson and Mittermeier 2009). Therefore, in agreement with the hypothesis of causal relationship between feeding specialization and taste receptor loss, the available evidence suggests that a feeding specialization, likely piscivory, triggered the processes of loss of the sweet and umami receptors over 8 million years ago in a common ancestor of the extant otters. Although these processes terminated in several lineages after no less than ~3 million years (loss of the sweet receptor in the *Enhydra lutris* lineage), no less than ~4.5 million years (loss of the sweet receptor in the *Lutra lutra* and *Amblonyx cinereus* lineages and the umami receptor in the *Lutra lutra* lineage), and no less than ~5.5 million years (loss of the sweet receptor in the *Lontra longicaudis* lineage and the umami receptor in the *Lontra longicaudis*, *Lontra canadensis*, *Amblonyx cinereus*, and *Aonyx capensis* lineages), they have not completed entirely, which is demonstrated by retention of the sweet receptor in *Lontra canadensis* (Fig. 3A) and the umami receptor in *Enhydra lutris* (Fig. 3B). However, given the anticipated low content of sweet and umami taste-eliciting compounds in the diets of *Lontra canadensis* and *Enhydra lutris*, it appears likely that the sweet receptor loss process is ongoing in *Lontra canadensis*, and the umami receptor loss process is ongoing in *Enhydra lutris*.

Situation similar to that in Lutrinae is found in the New World ictonychine crown clade Lyncodontini (molecularly dated to ~2.6–2.9 million years—Sato et al. 2012), where the sweet receptor has been lost in the lineage of *Lyncodon patagonicus* but is retained in the lineage of *Galictis cuja* (Fig. 3A). The apparently low content of sweet taste-eliciting compounds in the diets of *Galictis cuja* and the remaining (not evaluated here) extant species of Lyncodontini, *Galictis vittata* (Wilson and Mittermeier 2009), suggests that the process of loss of the sweet receptor in Lyncodontini has not been completed yet and is ongoing in both *Galictis* species. The convergent ongoing processes are likely to occur in Mustelinae, where *Mustela nudipes*, *Mustela nivalis*, and *Mustela furo* possess the sweet receptor despite their carnivorous habits (Fig. 3A).

## Conclusions

The sweet and umami taste receptor evolution in Carnivora demonstrates the prevailing correspondence between these receptors’ absence/presence and feeding habits and modes that is consistent with predictions of the hypothesis of causal relationship between feeding specialization and taste receptor loss. The 6 mismatches found between feeding specializations and sweet/umami receptor presence do not contradict this hypothesis because they can be explained by the stochastic and time-dependent nature of sweet/umami receptor loss process and the potential decelerating effect of selective pressure arising from TAS1R extragustatory functions, with the result that the sweet/umami receptor loss process has not been completed yet and is ongoing in each of these 6 species. Therefore we conclude that the carnivoran sweet and umami receptor evolution supports the idea that feeding specialization leads to taste receptor loss and is the main if not only triggering factor for evolutionary loss of taste receptors in vertebrates.

To universalize Jiang et al.’s (2012a) hypothesis and remove potential ambiguity from its original wording, we propose to reword “loss of taste receptor function in mammals is directly related to feeding specializations” (Jiang et al. 2012b) to “the evolutionary loss of taste receptors is driven by feeding specialization.” While this hypothesis proposes feeding specialization to be the factor that triggers a process of taste receptor loss, we propose that this process may be accelerated by abiotic environmental conditions or decelerated or even stopped by selective pressure resulting from extragustatory functions of taste receptor proteins.

We also clarify misunderstanding about mismatches between feeding specializations and taste receptor presence/absence (Zhao and Zhang 2012). As long as such mismatches involve taste receptor retention, they are not unexpected and do not necessarily contradict the hypothesized causal relationship between feeding specialization and taste receptor loss. This is because of the time dependence of taste receptor loss process and the existing potential for decelerating or blocking effect of extragustatory functions of taste receptor proteins. Therefore, one should not expect perfect correspondence between a feeding specialization and absence of the affected taste receptor at any one moment in evolutionary time and specifically before completion of the process of loss of that receptor.

What could contradict the hypothesized causal relationship between feeding specialization and taste receptor loss is absence of a taste receptor from a species whose feeding habit and mode do not deprive of the benefit of using the gustatory function of that receptor. However, even such mismatches are not unexpected and do not necessarily contradict this hypothesis if the absence of a taste receptor is actually inherited from the ancestor with a feeding specialization that favors loss of that receptor. Specifically, we mean situations where the process of loss of a taste receptor was triggered by feeding specialization and subsequently terminated in loss of that receptor in ancestors of a species whose feeding habit and mode favor presence of that receptor. For example, the sweet taste receptor was lost by a likely carnivorous avian ancestor and is therefore absent from all extant birds (Baldwin et al. 2014). Despite this loss there are multiple songbird species whose diets are rich in sugars. Studies have shown that these species and other extant songbirds can detect sweet taste-eliciting compounds using the ancestral umami taste receptor that was repurposed early in songbird evolution to function as a carbohydrate receptor (Baldwin et al. 2014; Toda et al. 2021).

## Supplementary Material

bjac033_suppl_Supplementary_Material

## Data Availability

DNA data accession information is in this article and Supplementary Tables S1 and S2.
