# Supplementary material for: Role of feeding specialization in taste receptor loss: insights from sweet and umami receptor evolution in Carnivora
Source: Chem Senses. 2022 Nov 22;47:bjac033. doi: 10.1093/chemse/bjac033 (PMC9680018; doi:10.1093/chemse/bjac033)
Supplement: bjac033_suppl_Supplementary_Material [file bjac033_suppl_supplementary_material.pdf]

Chemical Senses
Supplementary Data

Role of feeding specialization in taste receptor loss: insights from sweet and umami receptor evolution in Carnivora

Mieczysław Wolsan[1] and Jun J. Sato[2]

[1]Museum and Institute of Zoology, Polish Academy of Sciences, Wilcza 64, 00-679 Warszawa, Poland
[2]Department of Biotechnology, Fukuyama University, Higashimura-cho, Aza, Sanzo, 985-1, Fukuyama 729-0292, Japan

Contents

Table S1: Origin and accessibility of DNA sequences generated in this study 2
Table S2: Information on DNA sequences retrieved from DDBJ/ENA/GenBank 4
Table S3: Primers used to amplify exon 1 of *TAS1R1* 5
Table S4: Primers used to amplify exon 2 of *TAS1R1* 5
Table S5: Primers used to amplify exon 3 of *TAS1R1* 6
Table S6: Primers used to amplify exon 4 of *TAS1R1* 7
Table S7: Primers used to amplify exon 5 of *TAS1R1* 8
Table S8: Primers used to amplify exon 6 of *TAS1R1* 9
Table S9: Primers used to amplify exon 1 of *TAS1R2* 10
Table S10: Primers used to amplify exon 2 of *TAS1R2* 10
Table S11: Primers used to amplify exon 3 of *TAS1R2* 10
Table S12: Primers used to amplify exon 4 of *TAS1R2* 10
Table S13: Primers used to amplify exon 5 of *TAS1R2* 11
Table S14: Primers used to amplify exon 6 of *TAS1R2* 11
Table S15: Primers used to amplify exon 1 of *TAS1R3* 11
Table S16: Primers used to amplify exon 2 of *TAS1R3* 12
Table S17: Primers used to amplify exon 3 of *TAS1R3* 13
Table S18: Primers used to amplify exon 4 of *TAS1R3* 14
Table S19: Primers used to amplify exon 5 of *TAS1R3* 14
Table S20: Primers used to amplify exon 6 of *TAS1R3* 15
Figure S1: Evolution of the sweet and umami taste receptors in Carnivora in relation to feeding habits and modes 16
References 17

Table S1. Origin and accessibility of DNA sequences generated in this study

Species	Voucher specimen, tissue sample, and/or DNA sample		DDBJ/ENA/GenBank accession		
	Reference number(s)	Geographic origin	*TAS1R1*	*TAS1R2*	*TAS1R3*
Ailurus fulgens	JS 191	Asa Zoological Park, Hiroshima, Japan (no wild locality)	LC727905	LC654483	LC654495
Amblonyx cinereus	MZB 24297, JS 190	Jawa, Indonesia	LC727906	LC654484	LC727947
Arctonyx collaris	YP 6001, JS 212	Xishuangbanna, Yunnan, China	LC727907	—	LC727948
Canis lupus	JS 230	Hruskie, Augustów Forest, Poland	LC727908	—	LC727949
Cerdocyon thous	GD 515, JS 272	Central, Paraguay	LC727909	—	LC727950
Enhydra lutris	TH 257, JS 122	Alaska, USA	LC727910	—	LC727951
Galictis cuja	MC 795, JS 270	42°28'10" S, 64°22'06" W, Valdes Peninsula, Chubut, Argentina	—	LC654485	LC654496
Gulo gulo	TH 150, JS 193	Sakhalin, Russia	LC727911	—	LC727952
Ictonyx striatus	JS 678	No locality	LC727912	—	LC727953
Lontra canadensis	TH 332, JS 162	Canada	LC727913	—	LC654497
Lontra longicaudis	MFA-ZV-MH 2, JS 273	Cayastá, Santa Fé, Argentina	LC727914	LC654486	LC727954
Lutra lutra	JS 331	Wetlina, Bieszczady Mountains, Poland	LC727915	LC654487	LC727955
Lycalopex gymnocercus	JS 326	Buenos Aires, Argentina	LC727916	—	LC727956
Lyncodon patagonicus	MC 379, JS 266	Puerto Madryn, Chubut, Argentina	—	LC654488	—
Lynx lynx	JS 328	Białowieża Forest, Poland	LC727917	—	LC727957
Martes americana	TH 329, JS 150	Indian Creek, Whiteshell Provincial Park, Manitoba, Canada	LC727918	—	LC727958
Martes foina	JS 320	Białowieża, Poland	LC727919	—	LC727959
Martes melampus	TH 358, JS 218	Tokushima, Shikoku, Japan	LC727920	LC654489	LC654498
Martes zibellina	TH 401, JS 228	Teshio, Hokkaido, Japan	LC727921	—	LC727960
Meles anakuma	KT 2996, JS 12	Miyazaki, Kyushu, Japan	LC727922	LC654490	LC654499
Mellivora capensis	SU HB30, JS 239	25°37'58" S, 20°34'57" E, Kgalagadi Transfrontier Park, South Africa	LC727923	—	—
Melogale moschata	AK 703, JS 95	Vietnam	LC727924	—	—
	TH 322, HS 2372, JS 143	Taichung County, Taiwan	LC727925	—	LC727961
Melursus ursinus	HS 1421, JS 323	Nogeyama Zoological Gardens, Yokohama, Japan (no wild locality)	LC727926	—	LC727962
Mephitis mephitis	HTS 3, JS 803	Obihiro Zoo, Japan (no wild locality)	LC727927	LC654491	LC654500
Mustela altaica	AK 805, JS 106	Cherga, Altai Republic, Russia	LC727928	—	LC727963
Mustela erminea	TH 324, JS 145	Canada	LC727929	—	LC727964
Mustela kathiah	TH 321, JS 155	Kunming, Yunnan, China	LC727930	—	LC727965
Mustela lutreola	AK 13, JS 41	Novosibirsk, Russia	LC727931	—	LC727966
Mustela nivalis	SY 120, JS 795	Otoineppu, Hokkaido, Japan	LC727932	LC654492	LC654501
Mustela putorius	JS 797	Białowieża, Poland	LC727933	—	LC727967
Mustela sibirica	TH 446, JS 280	Primorsky Krai, Russia	LC727934	—	LC727968
Mustela strigidorsa	ANWC M32057, HS 4042	Oudomsouk, Nakai, Khammouan, Laos	LC727935	—	LC727969
Mydaus javanensis	JS 229	Mount Salak, Bogor, Java, Indonesia	LC727936	—	LC727970
Neogale vison	TH 445, JS 279	Primorsky Krai, Russia	LC727937	—	LC727971
Nyctereutes procyonoides	JS 235	Urwitałt, Mikołajki, Poland	LC727938	—	LC727972

(*Continued*)

Table S1 (*Continued*)

Species	Voucher specimen, tissue sample, and/or DNA sample		DDBJ/ENA/GenBank accession		
	Reference number(s)	Geographic origin	*TAS1R1*	*TAS1R2*	*TAS1R3*
Paguma larvata	TH 113, JS 806	Ehime, Shikoku, Japan	LC727939	—	—
Paradoxurus hermaphroditus	JS 805	Myanmar	LC727940	—	—
Pekania pennanti	TH 331, JS 152	Manitoba, Canada	LC727941	—	LC727973
Procyon cancrivorus	MFA-ZV-MH 17, JS 327	Garay, Santa Fé, Argentina	LC727942	—	—
Taxidea taxus	TH 334, JS 182	Napinka, Manitoba, Canada	LC727943	—	LC727974
Ursus thibetanus	AK 740, JS 324	Beltsovo, Primorsky Krai, Russia	LC727944	LC654493	LC654502
Vormela peregusna	IPEE 345, JS 269	Lake Sevan region, Armenia	LC727945	—	LC727975
Vulpes vulpes	JS 234	Różan, Maków Mazowiecki, Poland	LC727946	LC654494	LC654503

AK, Alexey Kryukov's collection deposited in the Institute of Biology and Soil Science, Russian Academy of Sciences, Vladivostok, Russia; ANWC, Australian National Wildlife Collection, Commonwealth Scientific and Industrial Research Organisation, Canberra, Australia; GD, Guillermo D'Elía's collection deposited in the Museo de Historial Natural de Paraguay, Asunción, Paraguay; HS and HTS, Hitoshi Suzuki's collection deposited in the Graduate School of Environmental Science, Hokkaido University, Sapporo, Japan; IPEE, Animal Tissue Depository for DNA Analysis, A. N. Severtsov Institute of Ecology and Evolution, Russian Academy of Sciences, Moscow, Russia; JS, Jun Sato's collection deposited in the Department of Biotechnology, Fukuyama University, Japan; KT, Kimiyuki Tsuchiya's collection deposited in the Graduate School of Environmental Science, Hokkaido University, Sapporo, Japan; MC, Marcelo Carrera's collection, Puerto Madryn, Argentina; MFA-ZV-MH, Área Zoología Vertebrados, Museo Provincial de Ciencias Naturales "Florentino Ameghino," Santa Fé, Argentina; MZB, Museum Zoologicum Bogoriense, Indonesian Institute of Sciences, Cibinong, Indonesia; SU, Department of Botany and Zoology, Stellenbosch University, Stellenbosch, South Africa; SY, Shumpei Yasuda's collection deposited in the Department of Biotechnology, Fukuyama University, Japan; TH, Tetsuji Hosoda's collection deposited in the Department of Biotechnology, Fukuyama University, Japan; YP, Ya-Ping Zhang's collection deposited in the Kunming Institute of Zoology, Chinese Academy of Sciences, Kunming, China.

Table S2. Information on DNA sequences retrieved from DDBJ/ENA/GenBank

Species	DDBJ/ENA/GenBank accession		
	TAS1R1	*TAS1R2*	*TAS1R3*
Ailuropoda melanoleuca	—	GCA_000004335.1	—
Ailurus fulgens	—	—	LNAC01000817
Amblonyx cinereus	—	JN130352[a]	—
Canis familiaris	GCA_000002285.2	GCA_000002285.2	GCA_000002285.2
Enhydra lutris	GCA_002288905.2	GCA_002288905.2	GCA_002288905.2
Felis catus	GCA_018350175.1	—	—
Lutra canadensis	—	JN130357	—
Martes flavigula	—	LC425460	—
Martes martes	—	LC425461	—
Mustela itatsi	—	LC425463	—
Mustela nudipes	—	LC425464	—
Procyon lotor	—	LC425470	LC425487
Ursus maritimus	—	GCA_000687225.1	—

[a]Only exon 3.

Table S3. Primers used to amplify exon 1 of *TAS1R1*

Name	Sequence (5' to 3')	Source
T1R1_EX1_F1	GGCCATGCCAGGCACAGGAC	Reference 1
T1R1_EX1_R1	CCCCTCACTCACCTGTCACAGAGRGT	Reference 1
Tas1r1_ex1_CanidR1	GCAGAGGTCCTTCTAGGCACAG	This study
Tas1r1_ex1_dogpandaF1	CTCTGCTCGGCCATGCCAGGCA	This study
Tas1r1_ex1_dogpandaR1	CCCCAGAGGCAGAAAGGGCA	Reference 2
Tas1r1_Ex1_Fw2	GGCCAGCATGTCACTCCTGGCAGCT	This study
Tas1r1_ex1_GuloF1	GCCAGGCACAGGAGCGTCTGGCC	This study
Tas1r1_ex1_GuloF2	GAGAGCTCTGCTCGGCCATGCC	This study
Tas1r1_ex1_GuloR1	CCGGCAGAGAGACTTTTAGGCAC	This study
Tas1r1_ex1_GuloR2	TAGGCACAGGACCCCCCACTCAC	This study
Tas1r1_ex1_GuloR3	CAGTCACCGTGGGCCTGCGTCT	This study
Tas1r1_ex1_GuloR4	CCGGACAGTGAGAGTGCACA	This study
Tas1r1_ex1_intCanidF1	GCCACAACACAGAGTCATCTCC	This study
Tas1r1_ex1_intMustIctF1	TGCTGCTGGGCCCTCAGCTGCCAC	This study
Tas1r1_ex1_intMustIctF2	CCTGGCAGCTCACCTGGTCAGCC	This study
Tas1r1_ex1_MustelaF1	CTCTGCTCGGCCATGCCAGGCG	Reference 2
Tas1r1_ex1_MustelaF2	GAAATTGGGAAGCATCTGGGCGGC	This study
Tas1r1_ex1_MustelaR1	TGGCGCCAGGGAAGGTCTAGCC	Reference 2
Tas1r1_ex1_MustIctR1	GCGAGGGACTGGAGGTGGTTCA	This study
Tas1r1_ex1_MustIctR2	TGAAGGTGGTGGCTGTAGGATGG	This study
Tas1r1_Ex1_Rv2	CACCGTGGGCCTGCGTCTCACC	This study

Table S4. Primers used to amplify exon 2 of *TAS1R1*

Name	Sequence (5' to 3')	Source
T1R1_EX2_F1	GCTCTCAGCYKGGCTTTCTCYACAG	Reference 1
T1R1_EX2_R2	CACAGGCACCAGAAACGGGCTCA	Reference 2
Tas1r1_ex2_CanidIntF1	GAGGAGATCAACAACTCCAC	This study
Tas1r1_ex2_dogF1	AAGAGGGTTTCGGCAAGGCCTTGC	This study
Tas1r1_ex2_dogR1	GCAGGGCCTCCTGCAATGTGAC	This study
Tas1r1_ex2_intMusteloideaF1	GGCCATGGCTACCACCTCTTCC	This study
Tas1r1_ex2_MustelaPinniR1	CCCCCTGCAATGTGACTCA	This study
Tas1r1_ex2_MustelaR1	GCAGAACCCCCTGCAATGTGAC	This study
Tas1r1_ex2_MustelidaeIntF1	GACGTGTGCTCCGAGTCGGCCAA	This study
Tas1r1_ex2_MusteloidIntF1	CTGCCCTGGCGGTGATTGGGCCTG	This study
Tas1r1_ex2_MusteloidIntF2	GCTACCACCTCTTCCAGGCCATG	This study

Table S5. Primers used to amplify exon 3 of *TAS1R1*

Name	Sequence (5' to 3')	Source
Tas1r1_ex3_A_colF2	CATTGCCTTCAAGGACATCATAC	This study
Tas1r1_ex3_A_colR2	CTCGGAGCAAGGCGCATGGGC	This study
Tas1r1_ex3_AonyxF1	CTGAACTTGCCGAACGGAACCC	This study
Tas1r1_ex3_CanidF1	TTGGCACAGTGTTGGGCGTGG	This study
Tas1r1_ex3_CanidR1	TCTGCCCCCAACACTTAGTTGGGC	This study
Tas1r1_ex3_CaniformF1	TTGAGACTGATGCCACTGAAC	This study
Tas1r1_ex3_ermineF1	GCTCTGAATTTCCAGAGGGACGG	This study
Tas1r1_ex3_ermineR1	AGGTTGCCAGGGGCTGGGCTCCCTTA	This study
Tas1r1_ex3_FelidF1	TTGGCACGGTGCTGGGTGTGG	This study
Tas1r1_ex3_Fw1	GGAGTGAAGCGGTATTACCC	Reference 10
Tas1r1_ex3_Fw2_5	GACTGGGCCATCTCCAGATA	Reference 2
Tas1r1_ex3_GulF1	GCTCTGAATTTCCAGATGGGCGG	This study
Tas1r1_ex3_GuMeIcLuF1	TGTGCCTCTGGAGCCTGTTCCAGGG	This study
Tas1r1_ex3_intCaniformR1	GCAATGCAGATGCCCTGCTGGG	This study
Tas1r1_ex3_intLutIctMustR1	CCTCTTCAAACTCCTTCAGGCC	This study
Tas1r1_ex3_intMusteloideaF1	CACGGTGCTGGGTGTGGCCATCC	This study
Tas1r1_ex3_intPinUrsF1_last	TGGGCCATCTCCAGACACATCAG	Reference 2
Tas1r1_ex3_intUrsidF3	CTGACGGCCAAGGTGTGGATCGC	This study
Tas1r1_ex3_intUrsidF4	GGTGGTCGTCTTCTCCAACAGGC	This study
Tas1r1_ex3_LutF1	GCTCTGAATTTCCAGATGGATGG	This study
Tas1r1_ex3_LutGuloR1	AGGGTGCCAGAGGCTGGGCTACCTTA	This study
Tas1r1_ex3_MusLutGulR1	CAGGTAATCACCCTGTTTCAGGG	Reference 2
Tas1r1_ex3_MusLutGulR2	GCTTGCCTCAGGTAATCACCCTG	This study
Tas1r1_ex3_MustelaCanidF1	CTGAACTTGCTGAACGGAACCCC	This study
Tas1r1_ex3_MustelaF1	TGTCAGCCTCCTATGTCCAGCGG	This study
Tas1r1_ex3_MustelidaeR1	GTGAAGCATCATGGCCTGCATC	This study
Tas1r1_ex3_MusteloideaR1	AAGTGAAGCCTAAGAGAGGA	This study
Tas1r1_ex3_MusteloideaR2	CTGAGAGGAAGCTTCTAACAAAGG	This study
Tas1r1_ex3_MusteloidPinniR1	GGCCAGCTGCCTGCTAGAGAA	Reference 2
Tas1r1_ex3_MustF1	ATTGGCACGGTGCTGGGTGTGGCCAT	This study
Tas1r1_ex3_PinniF1	CTGAACTTGCTGAACTGAACCCC	Reference 2
Tas1r1_ex3_PinniR1_last	TGCTTGCCTCAGGAAAGCACCC	Reference 2
Tas1r1_ex3_Rv1	GGCCTCTTCAAACTCCTTCAGGC	Reference 10
Tas1r1_ex3_Rv2_2	CTGCCAGGGGTAGACTCGGT	Reference 2
Tas1r1_ex3_UrsusR1	TTTCAGGGTGCCAGGGTCTGGGT	This study
Tas1r1_ex3_UrsusR2	CCTCAAAAAAGCAGCCTGTTTCA	This study
Tas1r1_ex5_MustR2	CTCGCTGGTGCCCTTCAAGACAGT	This study

Table S6. Primers used to amplify exon 4 of *TAS1R1*

Name	Sequence (5' to 3')	Source
T1R1_EX4_F1	TATYTCAGCTTCTRGAGCAGATCCGY	Reference 1
T1R1_EX4_R1	TTACCTGGTTGTCCTYYCCGTGCC	Reference 1
Tas1r1_ex4_ArctoidF1	GGCAGCTCCCTGATCCCTGTTTA	Reference 2
Tas1r1_ex4_ArctoidR1	CTGCCCATAAGGCAGTCCCCT	This study
Tas1r1_ex4_CaniformF1	GGAGGGCTCTGAATTTCCAGATGG	This study
Tas1r1_ex4_CaniformR1	CATGTTGGCCTGGTACCTGGGA	This study
Tas1r1_ex4_GuloF1	CTCTGATCCCTGTTTATTTCAG	This study
Tas1r1_ex4_MustCaniF1	GGCCTCCAGTTCAGCTGGACAT	This study
Tas1r1_ex4_MustIctF1	GAGAAGGAACGGTCTTTGC	This study
Tas1r1_ex4_MustIctF2	GCCCCAGGCACTTGCTTC	This study
Tas1r1_ex4_MustIctR1	CCAGGCTCCAGGCTGCCCATAA	This study
Tas1r1_ex4_MustIctR2	TGTCCAGAGCATCCCCAGGCTC	This study
Tas1r1_ex4_OtaridR1_last	GTCCACAGTATCCTCAGGCTCC	This study
Tas1r1_ex4_PinniF1_last	CATCTATGGCAGCTCCCTGGTC	This study
Tas1r1_ex4_UrsidR1	CTTGTGAGTGACCATGTCTC	This study
Tas1r1_ex5_IctR1	CTCGCCGGTGCCCTTCARGACAGT	This study
Tas1r1_ex5_itatsiR2	GACCCTAGGAGTGCCCAGTCATC	This study
Tas1r1_ex5_itatsiR3	CCCCTCATCTGCTCTATAGACCC	This study

Table S7. Primers used to amplify exon 5 of *TAS1R1*

Name	Sequence (5' to 3')	Source
T1R1_EX5_F1	CAGAACACCTGTGGCTTCTTGCAGGT	Reference 1
T1R1_EX5_R1	CACTCACCRCTCTTGTTGAGGAAGSTSC	Reference 1
Tas1r1_ex3_MustF1	ATTGGCACGGTGCTGGGTGTGGCCAT	This study
Tas1r1_ex5_ArctoidF1	CCAGGCCAACATGGCTCT	This study
Tas1r1_ex5_CanidF1	TCTGGGAGTGAGCTGTGAAGG	This study
Tas1r1_ex5_CanidF2	GGCAGATGCACAGAGACTATTTCC	This study
Tas1r1_ex5_CanidR1	CCTTACCCAGGTAGCTGCAGGCAAAG	This study
Tas1r1_ex5_caniformR1_last	GACCCTAGGAGTGCCCAGTC	This study
Tas1r1_ex5_caniformR2_last	AAGACATAGTGGGTCTGAGGA	This study
Tas1r1_ex5_GpandF1	TACTGTGGACACAGCTTACAC	This study
Tas1r1_ex5_IctR1	CTCGCCGGTGCCCTTCARGACAGT	This study
Tas1r1_ex5_intArctoidR1	CTGGTGCCCTTCAAGACAGTCG	This study
Tas1r1_ex5_itatsiF1	GGACACTCCCATGCTACCTGTGC	This study
Tas1r1_ex5_itatsiR1	CGCCCACTCTCTAGACACTCAC	Reference 2
Tas1r1_ex5_itatsiR2	GACCCTAGGAGTGCCCAGTCATC	This study
Tas1r1_ex5_itatsiR3	CCCCTCATCTGCTCTATAGACCC	This study
Tas1r1_ex5_MustelidaeF1	AGTCCGTGTGCTCCAGCGACTG	This study
Tas1r1_ex5_MustelidaeR1	GTGTGACCCAAGGGGGAT	This study
Tas1r1_ex5_MustF1	ACTGTCTGGAAGGGCACCAGCGGG	This study
Tas1r1_ex5_MustF2	ACTGTCTTGAAGGGCACCAGCGAG	This study
Tas1r1_ex5_MustIctF1_last	GTCATTGTGGGTTTCTACCAC	This study
Tas1r1_ex5_MustIctF2_last	GTGCCTAAGTCCGTGTGCTCCAG	This study
Tas1r1_ex5_MustIctF4	GCAGGTGCCTAAGTCCGTGTGC	This study
Tas1r1_ex5_MustR1	CCCGCTGGTGCCCTTCCAGACAGT	This study
Tas1r1_ex5_MustR2	CTCGCTGGTGCCCTTCAAGACAGT	This study
Tas1r1_ex5_OtaridF1_last	GCAGGTGCCCGAGTCTGTGTGC	Reference 2
Tas1r1_ex5_PinniCanidR2_last	GCTTCCTCAGAATTCAGTCTGG	Reference 2
Tas1r1_ex5_TrueMartenF1	CACCTGTGGCTTCTTGCAGGTG	This study
Tas1r1_ex5_UrsidF1	CCAGGCAGCACCCAGATGGCGAG	This study

Table S8. Primers used to amplify exon 6 of *TAS1R1*

Name	Sequence (5' to 3')	Source
T1R1_EX6_F1	CCTTTYCTTCCAGACCTCCACAGMTGC	Reference 1
T1R1_EX6_intF1	TACCAGCGCTTCCCTCAGCT	Reference 2
T1R1_EX6_intR1	AAAGGCGCTGACGGACAGGA	Reference 2
T1R1_EX6_R1	TCAGGTGGAGCCGCAGCGCC	Reference 1
Tas1r1_ex5_itatsiF1	GGACACTCCCATGCTACCTGTGC	This study
Tas1r1_ex6_ArctoidF1	GTAATTCATACAGGCATCTTG	Reference 2
Tas1r1_ex6_ArctoidR1	GTGCGTGGGAAGCAGGTTTCAC	Reference 2
Tas1r1_ex6_CanidF2	CCTTCCTCATTCCTGAACTGCT	This study
Tas1r1_ex6_CanidR2	GTGCGTAGGAAGCAGGATTCAC	This study
Tas1r1_ex6_CaniformiaF1	GCCAGCCTTGTGGGAAAGAAGA	Reference 2
Tas1r1_ex6_IctF1	ACCTCCACAGCTGCCAGCCTTGT	This study
Tas1r1_ex6_IctF2	AGGAGCCTTCTGGCTTTCCTTCCAG	This study
Tas1r1_ex6_IctR2	GGCCACTGAACTGGTGACCACA	This study
Tas1r1_ex6_intCanidF1	ACCCCGTTGCCCACCAGGGA	This study
Tas1r1_ex6_intCanidR1	ATGAAGCCCGGGGAGTTGGCCT	This study
Tas1r1_ex6_intIctMelF1	CTCAGCTGGTGGTGCTCGACTGC	This study
Tas1r1_ex6_intMelF1	GCTTCATGTTGGCTTTCGCCTAT	This study
Tas1r1_ex6_intMustelaF1	GCTTCCTGTTGGCTTTCGCCTAC	This study
Tas1r1_ex6_intMusteloideaF1	GCTTCATGTTGGCTTTCGCCTAC	This study
Tas1r1_ex6_intMusteloideaR1	CCCAAGAGATAGGTTCATGCCA	Reference 2
Tas1r1_ex6_intMustF1	TGACTTGGCTTGCGGTGTGGAC	This study
Tas1r1_ex6_intMustF2	GGCCCAGCTGCTCATCTGT	This study
Tas1r1_ex6_intMustR1	CCTTGCCCAGGTAGCTGCAGGCAAAG	This study
Tas1r1_ex6_intOtaroidR1	GGGAGTTGGCCTCTGTGCAAT	Reference 2
Tas1r1_ex6_intUrsidF1	GCTGGTGGTGCTTGATTGCACAG	This study
Tas1r1_ex6_intUrsidF2	CCCCACTGCCCACCAGGGAATA	This study
Tas1r1_ex6_intUrsidR1	GCCATTGTAGGCGAATGCCAAC	This study
Tas1r1_ex6_MustelidaeF1_last	CACTCCTGTTCTGCTCATCTAGC	This study
Tas1r1_ex6_MustelidaeR1_last	GTAGCTGCAGGCAAAGGCGCTGA	This study
Tas1r1_ex6_MustelidaeR2_last	GCTGGCCCTGCCCGCCTCCGCGG	This study
Tas1r1_ex6_MustelidaeR3_last	CGGAGGAGGTGTCAGGCCCGCC	This study
Tas1r1_ex6_MustGuloR1_last	TCCCTGGGCTGGCCCTGCCCGCCT	This study
Tas1r1_ex6_MustLutF1	CTCAGGAGCCTTCTGGCTTTCC	This study
Tas1r1_ex6_MustLutF2	CCTCACTCCTGTTCTGCTCATC	This study
Tas1r1_ex6_MustLutF3	GCAGGCCTATGTGGCCTCCCT	This study
Tas1r1_ex6_MustR1	CGCCTCGCTCGACCCGCGAC	This study
Tas1r1_ex6_PinniRedF1	CTCAGGAGCCTTCTGCCTTTCC	Reference 2
Tas1r1_ex6_UrsidR2	TCGTGGCCCTGCCCGCCTCAGT	This study

Table S9. Primers used to amplify exon 1 of *TAS1R2*

Name	Sequence (5' to 3')	Source
Tas1r2_ex1_CarniF1	AGCTGCCAGCTCTCTGATGAG	Reference 2
Tas1r2_ex1_MfuroR1	CCAGCCTCACATGGGAGACTCAC	This study

Table S10. Primers used to amplify exon 2 of *TAS1R2*

Name	Sequence (5' to 3')	Source
Tas1r2_ex2_MustF1	GCTTCCCACCCCACCCCATGTGG	This study
Tas1r2_ex2_MustPinniF1	GTTTTCCTATGKGAGGCAGAGGG	This study
Tas1r2_ex2_MustPinniF2	TCCCTTYCCCTTCTCACTCTG	This study
Tas1r2_ex2_MustPinniF3	AAAATGCCAGAATCTTCTGGAG	This study
Tas1r2_ex2_MustPinniR1	GTGAGCCCTCTGGACAACCACTC	This study
Tas1r2_ex2_MustPinniR2	GCTTCCTGCCTCCTCCTACCCCCAT	This study

Table S11. Primers used to amplify exon 3 of *TAS1R2*

Name	Sequence (5' to 3')	Source
Tas1r2_ex3F_intCarniR1	ACACGGCGCCCGTGAAGTTCTGG	This study
Tas1r2_ex3F_intCarniR2	ACCGGGTCGATGGCCCAGGACTC	This study
Tas1r2_ex3F_MfuroF1	GGCTGCCACGGTCAGCAGAGACC	This study
Tas1r2_ex3F_MfuroF2	TGGGGCGACAGTGGCCTGGGGAG	This study
Tas1r2_ex3L_AcinereF2	CCCGGGCTTCAGCGAGTTCCGC	This study
Tas1r2_ex3L_MfuroR1	ACTACCCACGGGCGGGGCCTCAC	This study
Tas1r2_ex3L_MustPinniF1	GCAGCTGTGGAAATCAAGGCTAG	This study

Table S12. Primers used to amplify exon 4 of *TAS1R2*

Name	Sequence (5' to 3')	Source
Tas1r2_ex4_MfuroR1	CCCGCAGCCATCGGAGAGCTGAC	This study
Tas1r2_ex4_MustPinniF1	GCAGCTGTGGAAATCAAGGCTAG	This study

Table S13. Primers used to amplify exon 5 of *TAS1R2*

Name	Sequence (5' to 3')	Source
Tas1r2_ex5_CarniF1_2	CTAGTTGGGCTCAGGGTCTC	This study
Tas1r2_ex5_CarniR2	GAGGGCTGTTCCTTCTCCCAC	This study
Tas1r2_ex5_CarniR3	GGGKGYRGGTCTGCAAGTCCTAC	This study
Tas1r2_ex5_CarniR4	CTACATGTRYGTGGAGTGGGGA	This study
Tas1r2_ex5_MfuroR1	GGGTGCAGGTCTGCAAGTCCTAC	This study
Tas1r2_ex5_MustF2	AGGTCCAGTTCTAATGGCAG	This study
Tas1r2_ex5_MustPinniF1	TCAGGCAGGCCAAACTAAGAGGG	This study
Tas1r2_ex5_MustPinniF2	CATCCCAGACAGGGGCCAGGC	This study
Tas1r2_ex5_MustR2	CTCCCTAACAGTGTGCACTGAGGG	This study

Table S14. Primers used to amplify exon 6 of *TAS1R2*

Name	Sequence (5' to 3')	Source
Tas1r2_ex6F_intMfuroR1	CCGTGAAGGACGCCACGAAGAC	This study
Tas1r2_ex6F_intMfuroR2	GCCTTGCGGTAGTTGCAGGACAG	This study
Tas1r2_ex6F_MfuroF1	AGCAAGGTGGGTGTGGCTGA	This study
Tas1r2_ex6F_MfuroF2	GAAGCTTCCCTGGGCTTTTGCAG	This study
Tas1r2_ex6L_intCarniF2	GCTTCACCATCTGCATCTCCTGC	This study
Tas1r2_ex6L_intCarniF3	TGGTCACCATCCTGGACCTCTTG	This study
Tas1r2_ex6L_MfuroR1	AAGCCTCCGGCCTCCTGGGCAGC	This study
Tas1r2_ex6L_MfuroR2	GGTGTCCCTGTGCCCAGCTGGGG	This study
Tas1r2_ex6M_intCarniF2	GCTTCACCATCTGCATCTCCTGC	This study
Tas1r2_ex6M_intCarniR3	CTGATGCCCAGAAGGTTGAG	This study

Table S15. Primers used to amplify exon 1 of *TAS1R3*

Name	Sequence (5' to 3')	Source
Tas1r3_ex1_ArctoidF1	AGGGGCCCCTGGGCTTGGCAG	Reference 2
Tas1r3_ex1_ArctoidF2	GATTTTGCTAAGCAAATCCTCTGC	Reference 2
Tas1r3_ex1_ArctoidF3	TCCCTGCTGGAAGCTGCCACCTGC	This study
Tas1r3_ex1_CanifF1	TCTCATTTGCCRTCCCTGCTGGAA	This study
Tas1r3_ex1_CarniR1onEx2	GGCCAGCGCCCAGAGCAGGCC	Reference 2
Tas1r3_ex1_MustelidaeF1	AAGCAAATCCTCTGCTCTCCCC	This study
Tas1r3_ex1_MustelidaeR1	ACACCCTGTCCCCACTC	This study
Tas1r3_ex1_MustelidaeR2	TGGGGCCACACAGGGCTGTGGG	Reference 2
Tas1r3_ex1_MusteloideaR1onEx2	GATCTCCTCCACGGCCATCTTCA	This study

Table S16. Primers used to amplify exon 2 of *TAS1R3*

Name	Sequence (5' to 3')	Source
Tas1r3_ex2_CaniformR1onEx3	TCTCCCGGTTGCTCAGCCGGTC	This study
Tas1r3_ex2_CarniF1onEx1	CCAATGCCACYGTGTGCACCAG	Reference 2
Tas1r3_ex2_CarniF2onEx1	GTGCTGGGYGGGCTCTTCCCCCT	This study
Tas1r3_ex2_CarniR1onEx3	TTGCTCAGCCGGTCGGTGCTGGC	Reference 2
Tas1r3_ex2_CarniR2onEx3	CCGTGCGGAAGAAGGAYGGGAAG	This study
Tas1r3_ex2_CaUrMuF1onEx1	CCCAATGCCACCGTGTGCACCAG	This study
Tas1r3_ex2_IctF1	TGTTCTTGAGTGGGGACAGGGT	This study
Tas1r3_ex2_LutGulF1	TGCTCCTGAGTGGGGACAGGGT	This study
Tas1r3_ex2_MustelaF1	GGCTCCCGAGTGGGGACAGGGT	This study
Tas1r3_ex2_MustelaF2	TCCCGAGTGGGGACAGGGT	This study
Tas1r3_ex2_MustelaR1	CTCCTGAGGTGGGGCCTCCTGCG	This study
Tas1r3_ex2_MustelidaeF2	TCCYGAGTGGGGACAGGGT	This study
Tas1r3_ex2_MustelidaeF2onEx1-2	GGTCTGGGCGGCAGGACACAAC	This study
Tas1r3_ex2_MustelidaeR2	GTAGCTGACCTGCARGGACAGCG	This study
Tas1r3_ex2_MustelidaeR2onEx3	CTGCAGGGACAGCGAGGCAGCT	This study

Table S17. Primers used to amplify exon 3 of *TAS1R3*

Name	Sequence (5' to 3')	Source
Tas1r3_ex3_CanidR1	CTGACACTGCTCCTCAGAAGTGC	This study
Tas1r3_ex3_CanifF1onEx2	GTTCTTCAGCTTCTTCCTCATGCC	Reference 2
Tas1r3_ex3_CanifF2onEx2	GCAAYTACACGCAGTACCAGCCCC	This study
Tas1r3_ex3_CaniformF1onEx2	GCAAGTTCTTCAGCTTCTTCCT	This study
Tas1r3_ex3_GulF1	CAGGAGCCCCCACCTCAGGAGC	This study
Tas1r3_ex3_intArctoidR1	CACCCACACCTTGGGCGAGAGCC	Reference 2
Tas1r3_ex3_intArctoidR2	GAGGTCAGCCAGGCCTCRCTGG	Reference 2
Tas1r3_ex3_intCarniF1	CTGCACCAGGTRAACCAGAGCAG	Reference 2
Tas1r3_ex3_intCarniF2	AGCAGYGTGCRGGTGGTGGTGCT	This study
Tas1r3_ex3_intCarniR1	TGCTGTAGCTGAAGAGGGTGCGG	Reference 2
Tas1r3_ex3_intCarniR2	CTGGCCACCCACACCTTGGG	This study
Tas1r3_ex3_intCaUrPiR1	AGCCAGGCCTCACTGGCCACCCA	This study
Tas1r3_ex3_intGulMelR1	AGAGCCTGCAGCGGATGCTGTAG	This study
Tas1r3_ex3_intLutGuloF1	AGGTGAACCAGAGCCGCGTGCAGG	This study
Tas1r3_ex3_intMusLutF2	GCTGGTGCTCTTCTCCTCCGCC	This study
Tas1r3_ex3_intMusteloideaF1	GGACCTGCTGCGCCAGGTGAACC	This study
Tas1r3_ex3_intMusteloideaF2	CTGCGCCAGGTGAACCAGAGCCG	This study
Tas1r3_ex3_intMusteloidR1	AGCCAGGCCTCGCTGGCCACCCA	This study
Tas1r3_ex3_intProcyonF1	CCTGCTGCACCAGGTCAACCAGA	This study
Tas1r3_ex3_intUrsidF1	TGCACCAGGTGAACCAGAGCAG	This study
Tas1r3_ex3_MusLutF1	TTCTTCCTCATGCCTCAGGTGTGC	This study
Tas1r3_ex3_MusLutF2	CCCGCAGGAGGCCCCACC	This study
Tas1r3_ex3_MustelaR1	CGGAAACTCCTCCTCCAGAGCAT	This study
Tas1r3_ex3_MustelidaeF1	AGCTGCCTCGCTGTCCCTGCAG	This study
Tas1r3_ex3_MustelidaeR1	KGGGGGCTCCCTGCCCTTAC	This study
Tas1r3_ex3_MustelidaeR1onEx3-4	CCTTACCTGCCAGGGCYGCACAG	This study
Tas1r3_ex3_MustelidaeR1onEx4	GCGTGGAAGGTCATGTTGTACAT	This study
Tas1r3_ex3_MusteloidR1onEx4	TGTTGTACATGTTCTCGAGGAG	This study
Tas1r3_ex3_UrsidR1	CTGAAATTCCTTCTCAGGAGTGC	This study
Tas1r3_ex3_UrsidR1onEx4	GTCATAATCCATGTGCACGTTCCC	This study

Table S18. Primers used to amplify exon 4 of *TAS1R3*

Name	Sequence (5' to 3')	Source
Tas1r3_ex4_AiMepF1onEx3	CACAACACGCTGCTCTGCAC	This study
Tas1r3_ex4_AiMepR1onEx5	CAGTCCACGCAGTCATAACAGCA	This study
Tas1r3_ex4_CanidF1onEx3	AACACACTGCTCTGCAATGCCTC	This study
Tas1r3_ex4_CanidR1onEx5	TAGCAGCAGGAGTGGAAGCC	This study
Tas1r3_ex4_CarniR1onEx5	CCRGGAGCACTGGGACACGGGC	This study
Tas1r3_ex4_CaUrMuF1onEx3	GTGTAYGGCGTGGCCCAGGCCCT	This study
Tas1r3_ex4_MeloF1onEx3	GGCCCTCCACAACACACTGCTCT	This study
Tas1r3_ex4_MusLutF1onEx3	CTGCTCTGCACTGCCTCAGGCTG	This study
Tas1r3_ex4_MusLutMeloR1onEx5	CCTTTCACTCGGCGCACCTGGCC	This study
Tas1r3_ex4_MustelaR1onEx5	CCGGGAGCACTGCGACACAGGC	This study
Tas1r3_ex4_MustelidaeR2onEx5	CAGCAGGARTGGAAGCCTTTC	Reference 2

Table S19. Primers used to amplify exon 5 of *TAS1R3*

Name	Sequence (5' to 3')	Source
Tas1r3_ex5_CanidF1	CAGCACAGCACAGCCTGAGCCCC	This study
Tas1r3_ex5_CanifF1onEx4	GCAGTCCCAGCTGTCCTGGCACA	Reference 2
Tas1r3_ex5_CarniR1onEx6	CACRCTGGGTGCAGAGGAGGTCA	Reference 2
Tas1r3_ex5_CarniR2onEx6	GGTCYGGGGACCACTGGTYCTG	Reference 2
Tas1r3_ex5_GulR1	GCTCATGCAGGCCTAGGAGAGC	This study
Tas1r3_ex5_GuMelR1	CAGAGTTGGCCTTGGAGCCAGGA	This study
Tas1r3_ex5_MustelaF1	CAGCCAAGCACAACCTGAGCCCC	This study
Tas1r3_ex5_MustelidaeF1	GGCCGCCTGAAGCTCTGGC	This study
Tas1r3_ex5_MustelidaeF1onEx4-5	CCCAGGAACAAGCTCTAGCTC	This study
Tas1r3_ex5_MustelidaeR1	CCACTKGCTCTGGCCTCAGAGT	This study
Tas1r3_ex5_UrMuR1onEx6	ACACTGGGTGCAGAGGAGGTCAT	This study
Tas1r3_ex5_UrsidF1onEx4	GTCCTGGCACACGCCAGGGAACC	This study

Table S20. Primers used to amplify exon 6 of *TAS1R3*

Name	Sequence (5' to 3')	Source
Tas1r3_ex6F_AiMepF1onEx5	TGCTGTTATGACTGCGTGGACTG	This study
Tas1r3_ex6F_ArctoidF1	GCTACCAGCGCAGCCCAGGT	This study
Tas1r3_ex6F_ArctoidF2	ACTCTGAGGCCAGAGCAAGTG	This study
Tas1r3_ex6F_CanifF1onEx5	TGCGTGGACTGCAAGGCCGGCA	This study
Tas1r3_ex6F_CanifF2onEx5	GGCTTCCRCTCCTGCTGTTA	Reference 2
Tas1r3_ex6F_CaUrF1onEx5	GGCAGCTATCAGCGCAGCCCAG	This study
Tas1r3_ex6F_intCaUrdR1	AAGGTGRAGCAGTGGCTGCTGGG	This study
Tas1r3_ex6F_intCaUrMuR1	AGTGGCTGCTGGGCCAGGCAGCT	This study
Tas1r3_ex6F_intMustelidaeR1	GGCTGCTGGGCCAGGCAGCTGGC	This study
Tas1r3_ex6F_intMustelidaeR2	AAGTGTGCTCAGGCAGCCAGT	This study
Tas1r3_ex6F_intMusteloidR1	GTGTGCTCAGGCAGCCAGT	This study
Tas1r3_ex6F_intMustR1	AATGTGGAGCAGTGGCTGCTGGG	This study
Tas1r3_ex6F_MustelidaeF1	ACTCTGAGGCCAGAGCAAGTGGG	This study
Tas1r3_ex6F_MustelidaeF2	CCCAGGCCTGCMTGAGCTGAGGA	This study
Tas1r3_ex6L_ArctoidR1	TCAGCAGCTGGGGTACAGTCTGG	Reference 2
Tas1r3_ex6L_ArctoidR2	AGTCARCYTGCAGTCAGGATC	This study
Tas1r3_ex6L_CanidR1	TCAGCAGCTGGGGTCCAGGTTGG	This study
Tas1r3_ex6L_CanidR2	GTTAGCCTACAGTTAGGATC	This study
Tas1r3_ex6L_intArctoidF1	GGTGACGGACTGGTGGGTGCTGC	Reference 2
Tas1r3_ex6L_intCanidF1	GGTGACAGACTGGTGGGTGCTAC	This study
Tas1r3_ex6L_intMustelidaeF1	GTGCGCTCCTGGGTCAGCTTC	This study
Tas1r3_ex6L_intUrsidF1	GACTGGTGGGTGCTGCCCACA	This study
Tas1r3_ex6L_MusIcLuR1	CATCGCAGCTAAGTCTGGGT	This study
Tas1r3_ex6L_MustelidaeR1	TTGCTTCATAGGAACTTGGTT	This study
Tas1r3_ex6L_UrsidR1	GGTACAGTCTGGGGGTTGCTTC	This study
Tas1r3_ex6M_intCanidF1	GGTGGCCCTGGGGCTCTTTAT	This study
Tas1r3_ex6M_intCarniF1	CTGCCTGGGCCTKGTCTGCCTCAG	This study
Tas1r3_ex6M_intCarniR1	GTGCCCAGGAAGCAGAGGARGGC	Reference 2
Tas1r3_ex6M_intCarniR2	GATCCAGGTGAYRAAGTAGG	Reference 2
Tas1r3_ex6M_intCaUrMuR1	CAGGAAGCAGAGGAAGGCCAGCA	This study
Tas1r3_ex6M_intCaUrPiF1	GGGCCACGGGCCTGCTTTGGC	This study
Tas1r3_ex6M_intMustelidaeF1	CCACTGGTTCAGGCCTCAGGTGG	This study
Tas1r3_ex6M_intMustelidaeF2	CCATGGGCCTGCTTTGGCCTGG	This study
Tas1r3_ex6M_intMustelidaeF3	CAGCGTCCTCCTGTTCCCTGGCC	This study
Tas1r3_ex6M_intMustelidaeR1	GGAAGGCCAGCACAGCATTGG	This study
Tas1r3_ex6M_intMustelidaeR2	CTGCACCAGGAAGGTGCCCAGG	This study
Tas1r3_ex6M_intMusteloidR1	GCACCAGGAAGGTGCCCAGGA	This study
Tas1r3_ex6M_intUrPiMuF1	GGCAGCCCTGGGGCTCTTCAT	Reference 2

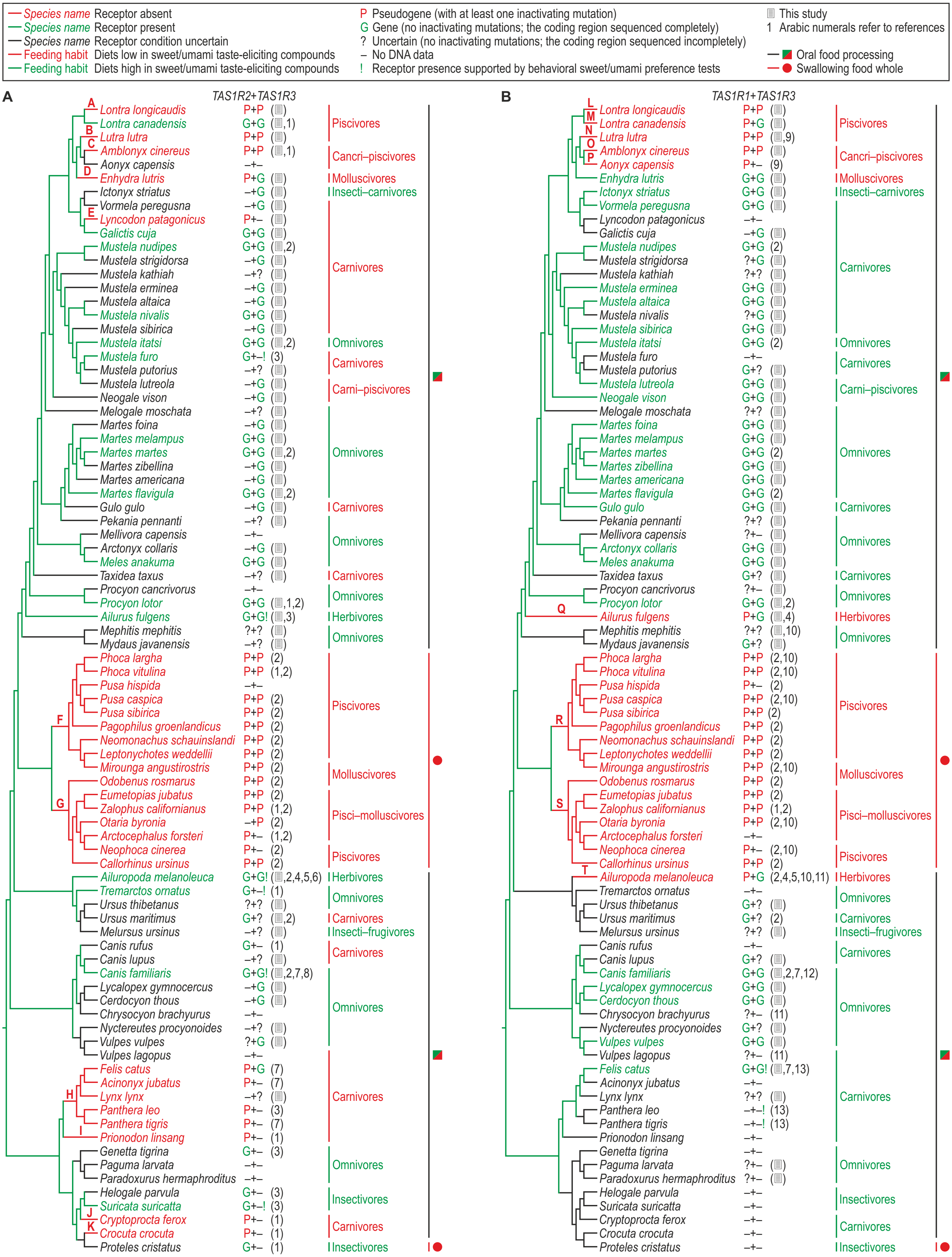

Figure S1. Evolution of the sweet (**A**) and umami (**B**) taste receptors in Carnivora in relation to feeding habits and modes. The events of loss of these receptors (A to T) are inferred from phylogenetic placement of *TAS1R* inactivating mutations (Tables 1 and 2). Phylogenies are compiled from references 2 and 14–21.

References

1. Jiang P, Josue J, Li X, Glaser D, Li W, Brand JG, Margolskee RF, Reed DR, Beauchamp GK. 2012. Major taste loss in carnivorous mammals. *Proc Natl Acad Sci USA*. 109:4956–4961.
2. Wolsan M, Sato JJ. 2020. Parallel loss of sweet and umami taste receptor function from phocids and otarioids suggests multiple colonizations of the marine realm by pinnipeds. *J Biogeogr*. 47:235–249.
3. Li X, Glaser D, Li W, Johnson WE, O'Brien SJ, Beauchamp GK, Brand JG. 2009. Analyses of sweet receptor gene (*Tas1r2*) and preference for sweet stimuli in species of Carnivora. *J Hered*. 100:S90–S100.
4. Hu Y, Wu Q, Ma S, Ma T, Shan L, Wang X, Nie Y, Ning Z, Yan L, Xiu Y, et al. 2017. Comparative genomics reveals convergent evolution between the bamboo-eating giant and red pandas. *Proc Natl Acad Sci USA*. 114:1081–1086.
5. Li R, Fan W, Tian G, Zhu H, He L, Cai J, Huang Q, Cai Q, Li B, Bai Y, et al. 2010. The sequence and de novo assembly of the giant panda genome. *Nature*. 463:311–317.
6. Jiang P, Josue-Almqvist J, Jin X, Li X, Brand JG, Margolskee RF, Reed DR, Beauchamp GK. 2014. The bamboo-eating giant panda (*Ailuropoda melanoleuca*) has a sweet tooth: behavioral and molecular responses to compounds that taste sweet to humans. *PLoS One*. 9:e93043.
7. Li X, Li W, Wang H, Cao J, Maehashi K, Huang L, Bachmanov AA, Reed DR, Legrand-Defretin V, Beauchamp GK, et al. 2005. Pseudogenization of a sweet-receptor gene accounts for cats' indifference toward sugar. *PLoS Genet*. 1:e3.
8. Ferrell F. 1984. Preference for sugars and nonnutritive sweeteners in young beagles. *Neurosci Biobehav Rev*. 8:199–203.
9. Tarusawa Y, Matsumura S. 2020. Comparative analysis of the umami taste receptor gene *Tas1r1* in Mustelidae. *Zool Sci*. 37:122–127.
10. Sato JJ, Wolsan M. 2012. Loss or major reduction of umami taste sensation in pinnipeds. *Naturwissenschaften*. 99:655–659.
11. Zhao H, Yang J-R, Xu H, Zhang J. 2010. Pseudogenization of the umami taste receptor gene *Tas1r1* in the giant panda coincided with its dietary switch to bamboo. *Mol Biol Evol*. 27:2669–2673.
12. Shi P, Zhang J. 2006. Contrasting modes of evolution between vertebrate sweet/umami receptor genes and bitter receptor genes. *Mol Biol Evol*. 23:292–300.
13. Beauchamp GK, Maller O, Rogers JG Jr. 1977. Flavor preferences in cats (*Felis catus* and *Panthera* sp.). *J Comp Physiol Psychol*. 91:1118–1127.
14. Sato JJ, Wolsan M, Prevosti FJ, D'Elía G, Begg C, Begg K, Hosoda T, Campbell KL, Suzuki H. 2012. Evolutionary and biogeographic history of weasel-like carnivorans (Musteloidea). *Mol Phylogenet Evol*. 63:745–757.
15. Koepfli K-P, Deere KA, Slater GJ, Begg C, Begg K, Grassman L, Lucherini M, Veron G, Wayne RK. 2008. Multigene phylogeny of the Mustelidae: resolving relationships, tempo and biogeographic history of a mammalian adaptive radiation. *BMC Biol*. 6:10.
16. Pagès M, Calvignac S, Klein C, Paris M, Hughes S, Hänni C. 2008. Combined analysis of fourteen nuclear genes refines the Ursidae phylogeny. *Mol Phylogenet Evol*. 47:73–83.
17. Lindblad-Toh K, Wade CM, Mikkelsen TS, Karlsson EK, Jaffe DB, Kamal M, Clamp M, Chang JL, Kulbokas EJ III, Zody MC, et al. 2005. Genome sequence, comparative analysis and haplotype structure of the domestic dog. *Nature*. 438:803–819.
18. National Academies of Sciences, Engineering, and Medicine. 2019. *Evaluating the Taxonomic Status of the Mexican Gray Wolf and the Red Wolf*. Washington (DC): National Academies Press.
19. Johnson WE, Eizirik E, Pecon-Slattery J, Murphy WJ, Antunes A, Teeling E, O'Brien SJ. 2006. The late Miocene radiation of modern Felidae: a genetic assessment. *Science*. 311:73–77.

20. Gaubert P, Cordeiro-Estrela P. 2006. Phylogenetic systematics and tempo of evolution of the Viverrinae (Mammalia, Carnivora, Viverridae) within feliformians: implications for faunal exchanges between Asia and Africa. *Mol Phylogenet Evol.* 41:266–278.
21. Eizirik E, Murphy WJ, Koepfli K-P, Johnson WE, Dragoo JW, Wayne RK, O'Brien SJ. 2010. Pattern and timing of diversification of the mammalian order Carnivora inferred from multiple nuclear gene sequences. *Mol Phylogenet Evol.* 56:49–63.
